# Should I Eat or Should I Go? Acridid Grasshoppers and Their Novel Host Plants: Potential for Biotic Resistance

**DOI:** 10.3390/plants7040083

**Published:** 2018-10-07

**Authors:** Alina Avanesyan

**Affiliations:** Department of Entomology, University of Maryland, 4291 Fieldhouse Drive, 3128 Plant Sciences, College Park, MD 20742, USA; alina@umd.edu; Tel.: +1-301-405-7877

**Keywords:** Acrididae, herbivory, introduced plants, invasive species, novel associations, plant-insect interactions

## Abstract

Novel, non-coevolved associations between introduced plants and native insect herbivores may lead to changes in trophic interactions in native communities, as well as to substantial economic problems. Although some studies in invasion ecology demonstrated that native herbivores can preferentially feed on introduced plants and therefore contribute to the biotic resistance of native communities to plant invasions, the role of acridid grasshoppers as native generalist insect herbivores is largely overlooked. This systematic review aimed to identify patterns of grasshopper feeding preferences for native versus introduced plants and, consequently, a potential of grasshoppers to provide biotic resistance of native communities. The analysis of 63 records of feeding preference trials for 28 North-American grasshopper species (retrieved from 2146 studies published during 1967–2017) has demonstrated a preference of grasshoppers for introduced host plants, and identified 12 preferred introduced plants with high or middle invasive ranks. A significant effect of the life stage (*p* < 0.001), but not the experimental environment, plant material, and measurements, on grasshopper preferences for introduced plants was also detected. Overall, results suggest a potential of acridid grasshoppers to contribute to the biotic resistance of native communities. The review also provides methodological recommendations for future experimental studies on grasshopper-host plant interactions.

## 1. Introduction

Human societies are increasingly moving plant species around the globe: Plants are introduced intentionally for food, landscape restoration, ornamental, and other purposes [1,2], as well as unintentionally, with imported plant material, crop seeds, commercial goods, soil, and by other pathways [1,3,4]. Some introduced plants escape cultivation and ornamental settings, disperse, and successfully establish in natural ecosystems, becoming potentially invasive (i.e., species causing ecological and economic problems). In particular, out of 25,000 plants introduced in the U.S., an estimated 5000 plant species escaped, invading approximately 700,000 ha of U.S. wildlife habitat per year and, in most cases, displacing native plant species [1]. The estimated costs associated with such introduced crop weeds and pasture weeds are $27.9 and $6.0 billion per year, respectively [5]. Substantial economic and environmental problems are also caused by some of the introduced (exotic) trees and shrubs, as well as aquatic weeds, which affect the natural habitat of animal species, alter nutrient cycles, and displace native vegetation [1]. 

A variety of hypotheses have been proposed to explain such successful establishment of exotic plants in the introduced range; Catford et al. [6] synthesized 29 hypotheses, which are commonly accepted in studies on plant invasion ecology, that focus on different ecological mechanisms. The mechanisms derived from these hypotheses and associated with plant defenses and herbivory include, but are not limited to, plant escape from their co-evolved herbivores (enemy release hypothesis; [7]), plant high competitive ability (evolution of increased competitive ability hypothesis; [8]), plant higher production of chemicals novel for the invaded community (novel weapons hypothesis; [9]), and a shift from high-cost to low-cost defense compounds (shifting defense hypothesis; [10]).

Although many studies have demonstrated that introduced plants can outcompete native plants [11,12,13,14,15], not all plant introductions are successful, and not all naturalized plants (i.e., adapted to the introduced range) become invasive. One of the main mechanisms that has been proposed to explain the “failure” of introduced plants to establish is biotic resistance in native communities (biotic resistance hypothesis; [16,17,18,19]). In general, the term, “biotic resistance”, describes “the ability of resident species in a community to reduce the success of exotic invasions” [20]. Thus, any effect, such as competition, parasitism, herbivory, or predation, from any native species can contribute to the biotic resistance of a community to its invaders. The biotic resistance hypothesis is particularly well explored in studies on invasive plants [20], and it predicts that native generalist herbivores will prefer to feed on introduced plants that do not share coevolutionary history with these native herbivores, and, therefore, will be less defended compared to native plants [18]. Following Maron and Vilà [18], many studies published in subsequent years demonstrated that exotic plants can be preferentially consumed in a plant’s introduced range by non-coevolved native herbivores [11,19,21,22,23,24,25,26,27,28]. Similar results on generalist herbivores’ preferences for exotic plants were also obtained from studies on non-insect invertebrates [29]. 

Many authors emphasized that by incorporating exotic plants into their diet, native generalist insect herbivores can contribute to the biotic resistance of native communities to plant invasions [19,22,25,27,28]. In this regard, orthopterans, which are the most abundant aboveground insects [30], and particularly grasshoppers, are especially unique study organisms for exploring the role of native generalist insect herbivores in the biotic resistance of native communities.

There are at least three characteristics of grasshoppers that make them especially beneficial for studies on invasion ecology. First, the most remarkable characteristic is that all North-American species of grasshoppers are native: No introduced species are known, in contrast to approximately 1500 species of other insects that have invaded North America over the past few hundred years [30]. Consequently, any association between a North-American grasshopper species and an introduced plant is a novel association—i.e., the association between resident (native) and non-resident (exotic) species, “in which at least one species has little or no experience with relevant ecological traits of its interaction counterpart” [31]. 

Secondly, almost all grasshopper species are herbivorous, and most of them are either polyphagous (able to utilize plants from more than one family) or oligophagous (able to feed on several genera of plants, which are restricted to one plant family) [30]. Thus, researchers can investigate the feeding responses from grasshoppers to a wide range of introduced plants—plants with different life forms, perenniality, etc., and presumably different herbivore resistance and tolerance traits. Finally, previous studies have shown that grasshoppers can utilize introduced plants [13,25,26,27,28,32], including highly invasive plants [33]. So, researchers can explore grasshopper feeding patterns on the introduced plants and grasshoppers’ potential for suppressing plant growth and preventing plant establishment in the introduced range. 

Additional advantages of using grasshoppers in feeding preference trials include: (a) They are relatively easy to collect and maintain, both short-term and long-term; (b) due to their mode of feeding (grazing) and relatively fast development (during one season), it is easy to detect and quantify their impact on plants (e.g., leaf damage), as well as estimate grasshopper consumption, growth, and performance; and (c) as in other hemimetabolous insects, host plant use in grasshopper nymphs and adults are often similar, so different developmental stages can be used in the same or similar feeding experiments. 

Despite these advantages, the use of grasshoppers as native generalist insects is largely overlooked in experimental studies on invasion ecology. Most studies on introduced plants and associated insect herbivory have primarily utilized either specialist insects [24], or generalist insects from Lepidoptera [15,21,34]. Many studies on grasshopper feeding preferences have been conducted primarily in relation to the nutritional quality of plants or an effect of plant abundance on grasshopper feeding [35]. A few studies have used grasshopper species in the experiments that involved feeding on native versus introduced plants [11,25,27,28,32,36,37]. The results from these studies, as well as methodological aspects, however, are inconsistent: The authors have used different plant parts (leaves, shoots, whole plants), different life forms (trees, shrub, grasses, etc.), C_3_ versus C_4_ plants, different measurements of grasshopper preferences for plants, etc. Consequently, little is known about the pattern (if any) of grasshopper feeding preferences on native versus introduced plants. 

This review focuses on host plant preferences of acridid grasshoppers (Orthoptera: Acrididae) on native versus introduced plants. Acrididae is one of the major orthopteran families with an estimated 630 species out of 1200 total orthopterans north of Mexico [30]. The main objectives of this systematic review are: (a) to identify patterns of grasshopper feeding preferences for native versus introduced plants and, consequently, grasshopper potential for biotic resistance of native communities (based on the findings from the published studies over the past 50 years); (b) to examine the invasive potential of preferred introduced host plants of grasshoppers (if any); and (c) to assess the effect of experimental conditions on grasshopper feeding choice. The review also identifies potential areas for future research and provides methodological recommendations for future experimental studies.

## 2. Results

### 2.1. Studies on Grasshopper Feeding Preferences over the Past 50 Years

The search for relevant scientific literature identified 2146 studies published between January, 1967 and September, 2017. Of those, 411 studies contained titles and abstracts relevant to insect herbivory, and only 87 studies were conducted on the North-American continent using North-American acridid grasshoppers. Final screening identified 13 studies that satisfied all the inclusion criteria developed for this review (Figure S1). Of these, only six studies explicitly compared grasshopper feeding on native versus introduced plant hosts, while the rest of the studies reported the diet preferences of grasshoppers without a focus on the plant origin. Most of the selected studies contained more than one record of grasshopper feeding preference trials. Thus, a total of 63 records of feeding preference trials for 28 North-American grasshopper species were identified and included in the analysis (Table S1). 

Based on the distribution of studies retrieved from the SpringerLink database (Figure S2a), the lowest number of published studies corresponds to the period of 1972–1974, and a substantial growth in research on the feeding of acridid grasshoppers is observed in the last 20 years: The annual number of publications reached its maximum in the period from 1998-2000 which was nine times higher compared to its minimum in 1972–1974. The distribution of published studies on grasshopper feeding on native and introduced plants, which were included in the analysis, indicated a substantial gap in such publications during 1974–1994 (Figure S2b).

**Figure 1:**
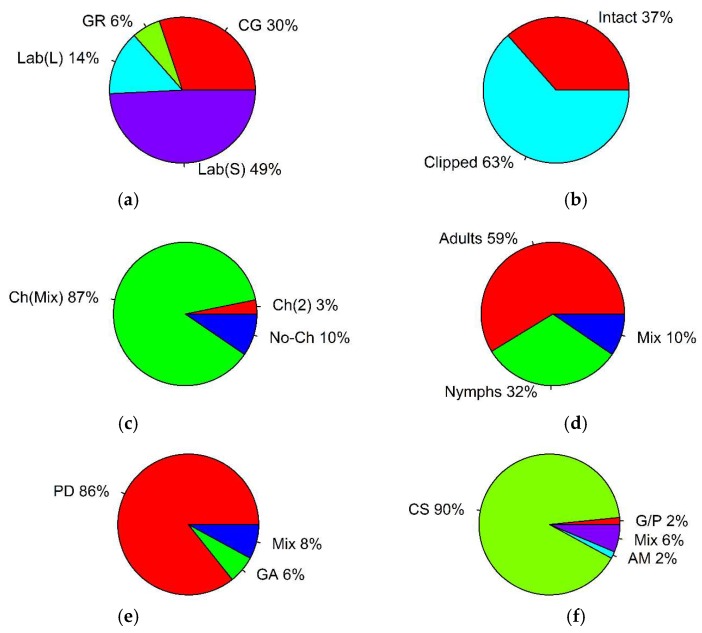
Percentage of studies that reported different experimental set-ups used to estimate grasshopper feeding preferences: (**a**) Types of the experimental environment used (CG: Common garden; GR: Greenhouse; Lab(S): Laboratory, clipped stems; Lab(L): Laboratory, clipped leaves); (**b**) type of plant material; (**c**) type of preference trial (Ch(Mix): Choice with a plant mixture; Ch(2): Choice with two plants; No-Ch: No-choice); (**d**) grasshopper life stage used; (**e**) general preference measurements (PD: Plant damage; GA: Grasshopper activity); and (**f**) grasshopper activity measurements (CS: Consumption; AM: Assimilation; G/P: Growth and performance).

Four different types of the experimental environment were identified from the selected feeding records (Table 1, Figure 1a): (1) Common garden experiment (intact plants); (2) greenhouse experiment (intact potted plants); (3) laboratory experiment with plant cuttings (a stem with several leaves); and (4) laboratory experiment with clipped plant leaves. The feeding records also revealed that various plant material (Figure 1b), preference trials (Figure 1c), grasshopper life stages (Figure 1d), general preference measurements (Figure 1e), and grasshopper activity measurements (Figure 1f) were utilized (see also Table 1). Feeding trials that used adult grasshoppers were prevalent (59% of all records; binomial test: *p* < 0.001). Also, choice feeding trials, when a mixture of plants was offered to grasshoppers to consume, were used more often (87% of all records; binomial test: *p* < 0.001). The analysis of the selected records also demonstrated that measurements of feeding preferences that applied to plant damage (86% of all records; binomial test: *p* < 0.001), as well as measurements of grasshopper consumption, were significantly prevalent (90% of all records; binomial test: *p* < 0.001) (Table 1). 

### 2.2. Grasshopper Feeding Preferences for Native Versus Introduced Plants

**Figure 2:**
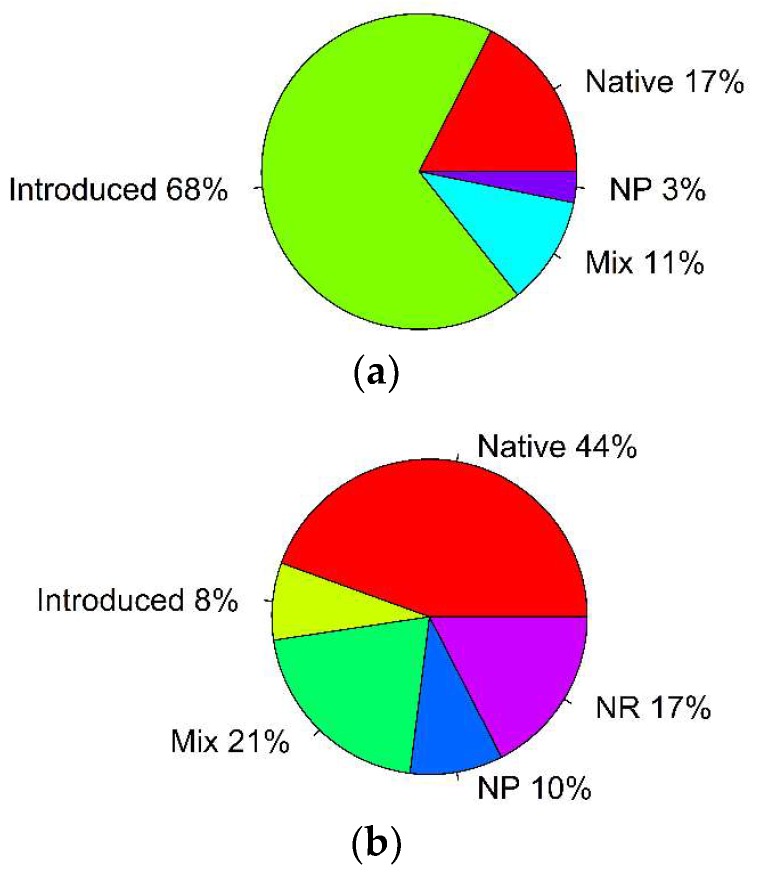
Percentage of studies that reported different outcomes of the feeding trials with acridid grasshoppers: (**a**) Most preferred plants; (**b**) least preferred plants (NP: No preferences observed; NR: Preferences not reported).

The analysis of grasshopper feeding preferences and an effect of different experimental conditions on grasshoppers’ most preferred plants revealed several interesting patterns. Introduced plants were prevalent among the most preferred plants (68% of all records; binomial test: *p* = 0.005; Table 1, Figure 2a), whereas native plants did not prevail among the least preferred plants (44% of all records; binomial test: *p* = 0.45; Table 1, Figure 2b).

Preference metrics were extracted from 55 studies, which were included in the meta-analysis (mean ± SE: 0.17 ± 0.22, Table S4). The studies demonstrated moderate heterogeneity, with Q = 94.75 (which was higher than 73.31, the critical value for 54 degrees of freedom according to the chi-square distribution) and I^2^ = 49%. Since the variability among the studies is presumably not only due to sampling error, a random effects model was used to estimate the effect of the experimental environment, plant material, grasshopper life stages, and measurement units on the outcomes from feeding trials. Following Neyeloff et al. [38], the weight of each study (W_v_) was adjusted with a following constant, v:

v = [Q − (sample size − 1)]/[∑(w) − (∑w^2^/∑w)], (1)

**Figure 3:**
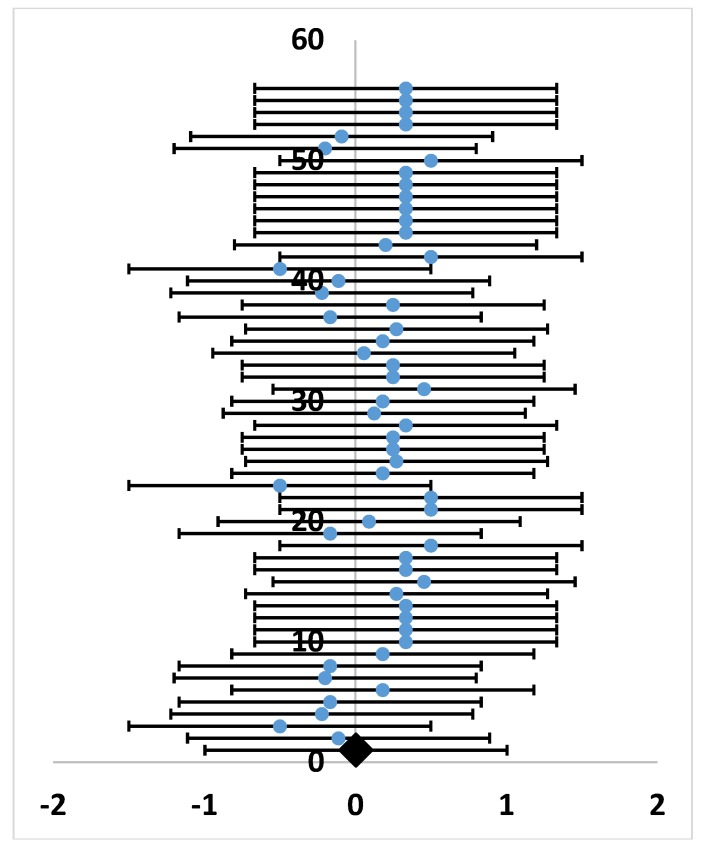
Forest plot of grasshopper preferences for introduced plants by study. Random effects model: (I^2^ = 0%). Blue dots and horizontal bars represent data for the preference metric and 95% confidence intervals, respectively. The black diamond represents the summary effect. The studies and effect sizes are presented on the Y-axis and X-axis, respectively. Outcomes, 95% confidence intervals, and sample sizes for 55 studies included in the meta-analysis are provided in the supplementary material (Tables S4 and S5).

W_v_ = 1/(SE^2^ + v), (2)

then, Q and I^2^–values were recalculated using adjusted W_v_ (Table S4). The results showed an acceptable Q = 51.71 and no observed heterogeneity (I^2^ = 0%) (Figure 3).

**Figure 4:**
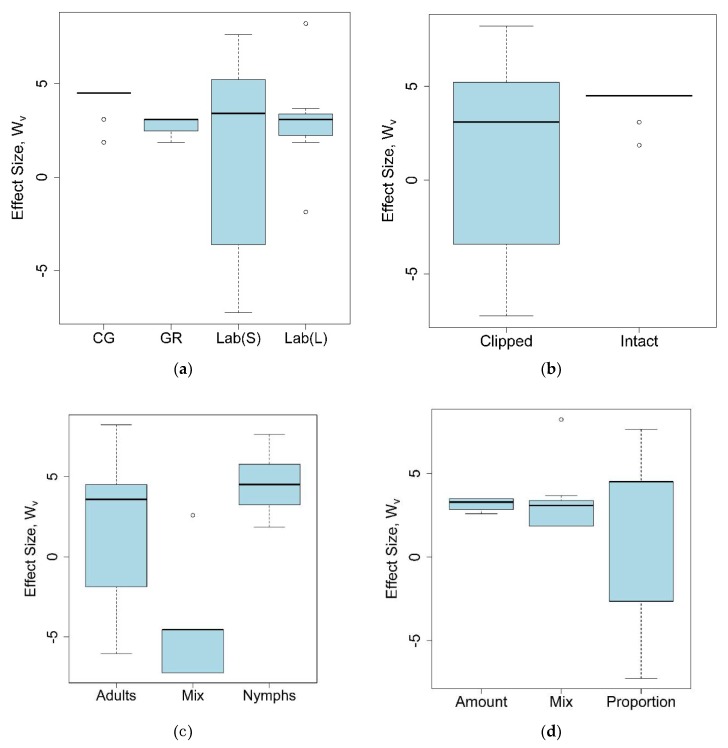
Mean effect sizes (with 95% confidence intervals) for studies conducting different feeding trials: (**a**) Studies used different experimental environments (CG: Common garden; GR: Greenhouse; Lab(S): Laboratory, clipped stems; Lab(L): Laboratory, clipped leaves); (**b**) studies used different plant material; (**c**) studies used different grasshopper life stages; (**d**) studies used different types of measurements (amount: Amount of plant tissue consumed by grasshoppers; proportion: Proportion of plant damage caused by grasshoppers). Outcomes and 95% confidence intervals for 55 studies included in the meta-analysis, as well as sample sizes, are provided in the supplementary material (Tables S4 and S5).

Among all the studied parameters, a significant effect of the grasshopper life stage only on the outcome of a feeding trial was observed (the Kruskal–Wallis test: χ^2^ = 13.96, d.f. = 2, *p* < 0.001). The effect size of using mixed life stages was significantly lower than that of using adults (the post hoc Nemenyi test: *p* = 0.014) or nymphs alone (the post hoc Nemenyi test: *p* < 0.001); whereas using either adults or nymphs alone did not affect the outcome of the feeding trial (the post hoc Nemenyi test: *p* = 0.17). All other parameters did not demonstrate a significant effect on grasshopper preferences for introduced plants (Figure 4a–d). 

### 2.3. Invasive Potential of Preferred Introduced Host Plants 

Among the most preferred and second preferred plants, across all the records, 22 introduced plants were identified; of these, two species (*Bromus inermis* and *Paspalum notatum*) have both introduced and native status on the North-American continent (Table S2). Twenty species were reported as “the most preferred” and two species as “the second preferred” for grasshoppers. Eleven species (50% of total identified plants) belong to the family, Poaceae, and are perennial and graminoid.

Invasive ranks were determined, when available, for 13 plant species. Of these, 12 species showed high or middle I-rank, Subrank III (Trends in distribution), and Subrank IV (managing difficulty), nine species showed high or middle Subrank I (ecological impact), and all 13 species showed high or middle Subrank II (current distribution). Species with reported high I-rank included *B. tectorum*, *Eichhornia crassipes*, and *Triadica sebifera*; and species with reported high/medium I-rank included: *B. inermis*, *Schedonorus arundinaceus*, and *Sorghum halepense*. Although medium I-rank was reported for *Miscanthus sinensis*, *Phleum pretense*, and *Poa pratensis*, these plants have high Subrank II (current distribution). Most grasshopper species were reported to have preferences for *B. inermis* (14 grasshopper species) and *Schedonorus arundinaceus* (nine grasshopper species) whereas other host plants were reported to be preferred by one grasshopper species and only *Phleum pretense* was reported to be preferred by four grasshopper species. 

The highest number of U.S. National Parks (18) and states (25) where the plant species were reported as invasive was seen for *Sorghum halepense*. *Bromus tectorum* has also been reported as invasive in a high number of U.S. National Parks (16), followed by *Dactylis glomerata* (10), *B. inermis* (eight), and *Taraxacum officinale* (seven). *Triadica sebifera* and *E. crassipes* were also reported as invasive in a high number of states (nine for both), followed by *M. sinensis* (eight), *Schedonorus arundinaceus* (seven), and *B. tectorum* (six). The rest of the plant species (if included) are currently on the invasive species list for 1–5 states and national parks.

## 3. Discussion

The primary goal of this review was to explore the feeding response of North-American acridid grasshoppers to introduced plants, as well as the grasshopper’s potential for biotic resistance of native communities. To achieve this goal, the patterns of grasshopper feeding preferences for native versus introduced plants, as well as the invasive potential of preferred introduced plants, were investigated based on the findings from experimental studies on grasshopper host plant preferences published during the past 50 years. 

An analysis of the retrieved literature showed a surprisingly low number of studies on acridid grasshopper host plant preferences that used both native and introduced plant species (13 out of 2146); of these, only six studies explicitly compared grasshopper feeding or performance on plants of different origin. This might be explained, in part, by the substantial economic importance of acridid grasshoppers due to their ability to outbreak; as a result, the focus of many experimental studies on the biology and ecology of grasshoppers has been often limited by grasshopper damage to different crop plants [39], grasshopper dietary selection based on plant quality [35,40], or their involvement in ecosystem nutrient cycling [41]. In the meantime, grasshopper feeding responses to introduced host plants and their contribution to the resistance of communities have remained largely unexplored. 

It is also possible, that, due to the focus of this review on grasshoppers’ feeding choices between native and introduced plants under the same environmental conditions, and, consequently, the use of relatively strict inclusion criteria, some of the relevant studies were not included. Particularly, a study on the feeding of three native *Schistocerca* species conducted by Otte [42], although analyzed in Parker and Hay [19], was not included in this review: Even though it involved native and introduced plants and a reported preference level for each plant, the study did not report grasshopper choice between native and introduced in each trial, and a small number of introduced plants (compared to that for natives) was used in the trials. Additionally, the author indicated that some plants were used in several trials while others were tested only once [42]. 

One of the main reasons for conducting this systematic review was to emphasize the advantages of using native acridid grasshoppers in invasion ecology and to encourage researchers to further explore grasshopper interactions with novel host plants. Consequently, in this discussion section, the (1) potential implications of the results for biotic resistance, (2) methodological recommendations for experimental design in future studies, and (3) suggested future research directions are focused on.

### 3.1. Potential Implications for Biotic Resistance 

Although <10% of insect herbivores are generalists [43], these insect species are promising candidates for consuming exotic host plants, and they can develop well on novel food compared to specialists [44]. Due to various morphological traits (such as stem height, plant architecture), as well as unique plant chemistry, introduced plants can be attractive for native insect herbivores, causing their host range expansion and even host plant shift. Such an ability to incorporate introduced plants in their diet has been demonstrated for many insect species outside of Orthoptera, such as *Pontia protodice* (southern cabbageworm) (Lepidoptera: Pieridae), *Pieris oleracea* (mustard white) (Lepidoptera: Pieridae), and *Pyrrharctia isabella* (the isabella tiger moth) (Lepidoptera: Erebidae) [44]. Although invasive plants can negatively impact native insects in many ways (see also Bezemer et al. [44]), some insect species can remain unaffected or even benefit from their novel host plants: Litt et al. [43], in their meta-analysis of the effects of invasive plants on arthropods, found that 17% of a total of 87 published studies reported an increase in the abundance, biomass, or richness of herbivores in response to plant invasion, and 26% of studies reported no changes. 

The main outcome from this systematic review was that the majority of studies demonstrated a preference of acridid grasshoppers for introduced host plants. Interestingly, there was no tendency for native host plants to be among the least preferred. The most remarkable outcome from the biotic resistance standpoint was that about 50% of the preferred introduced plants (for which the invasive rank was available) had a middle or high invasive rank. Particularly, *Bromus species* (*B. tectorum* and *B. inermis*) were among the most preferred introduced host plants. Both *B. tectorum* and *B. inermis* are highly invasive and competitive grasses. *Bromus tectorum* (European cheatgrass) has invaded and become well established on five million hectares in Idaho and Utah, causing a reduction in shrubs and other local vegetation, and affecting the habitats of some animal species [1]. Meanwhile, Cumberland et al. [33] showed that *Melanoplus bivittatus*, both nymphs and adults, exhibited a strong preference for *B. tectorium* over native plants (when preference was registered). *Bromus inermis* has also been reported to alter native vegetation and affect the population dynamics of native arthropods [45]; however, this review demonstrated that 50% of grasshopper species reviewed in this manuscript show a preference for *B. inermis* in various experimental settings [25,37,46,47].

An interesting finding from the meta-analysis (besides the methodological implications described below) was that apparently grasshopper adults and nymphs do not demonstrate significant differences in their preference for introduced plants. Such a feeding response to introduced, potentially invasive, plants may be especially critical in temperate regions where both grasshopper nymphs and adults uninterruptedly consume vegetation during the summer season. Due to their underdeveloped mouth parts, grasshopper nymphs easily consume young seedlings (rather than grown plants) and can feed on them as early as April or May, while adult grasshoppers are able to consume both the leaves and inflorescences of mature plants until late October. Consequently, as it was shown with exotic, potentially invasive, grasses [27,28], grasshoppers can affect the earlier establishment of plants in the introduced range in spring/early summer (as nymphs), as well as the growth of mature plants later in the season (as adults). Furthermore, given the polyphagous nature of both the nymphs and adults of most North-American acridid grasshoppers [48], they can cause damage to multiple introduced plant species, and potentially contribute to the biotic resistance of a community during the entire season.

Although this review showed that grasshoppers readily accept introduced plants and, in most feeding trials, exhibit a preference for introduced plants, the most effective candidate for biotic resistance is expected to have a potential to reduce the acreage of invasive plants, while its longevity and/or fecundity would remain unaffected. Only one study included in this review [25], however, addressed some of these issues, and apparently grasshopper density at a local scale might not be sufficient to completely suppress invader populations. Fielding and Conn [25] demonstrated that in experimental enclosures, *Melanoplus borealis* preferred to feed on *Crepis tectorium*, a novel, rapidly spreading invasive weed in Alaska, and at a high density, they significantly reduced the flower production and growth of *C. tectorium*. However, the authors mentioned that the grasshopper density used in the experiments was higher than their natural density, which could be one of the reasons why grasshoppers are not able to completely suppress *C. tectorium* populations in natural communities. 

In addition, the patterns detected in this review may or may not reflect the situation in nature, or at least not at all the locations. Only a few studies explored grasshopper preferences for introduced versus native plants under natural conditions. For example, Lankau et al. [36] and Siemann and Rogers [11] demonstrated that acridid grasshoppers did not avoid feeding on invasive *Sapium* plants in the field, however, the herbivory load was low. Avanesyan and Culley [28] previously demonstrated the prevalence of DNA of introduced plants in the gut contents of field collected *Melanoplus* grasshoppers. Future studies might further explore grasshopper consumption of native and introduced plants under natural field conditions. Litt et al. [43] also indicated that some generalist herbivores might prefer to feed on native plants because of high lignin and starch content in invasive plants. Interestingly, we previously demonstrated a preference of *Melanoplus* grasshoppers for *Miscanthus* plants, which possess high leaf toughness influenced by silica in their plant tissue [27]. Apparently, leaf toughness does not prevent grasshoppers from feeding on some of the invasive plants as opposed to such an effect on other herbivores. Future studies might focus on the effect of these factors on grasshopper responses to novel host plants.

### 3.2. Methodological Recommendations

A number of methodological studies was previously published on the design and analysis of preference trials [49,50]. Based on the results of this review, several additional methodological recommendations can be proposed specifically for the experimental set-up of future studies on grasshopper feeding preferences on native versus introduced host plants. 

#### 3.2.1. Using a Combination of Choice and No-choice Feeding Trials

A substantial number of studies (90%) analyzed in this review primarily used choice feeding trials with plant mixtures. While choice experiments undoubtedly provide information about grasshopper preferences for a host plant, they do not allow a researcher to easily assess grasshopper food assimilation on different plants. Meanwhile, information about how much food was taken and assimilated is critical for concluding whether a plant is suitable for grasshoppers and, consequently, can be utilized as a food plant. Such confirmation of grasshopper food consumption is also imperative for making predictions about the effect of native grasshoppers on introduced plant populations. To date, many studies have developed and used rapid methods of grasshopper diet confirmation and food utilization using molecular biology techniques [28,51,52,53,54]. Combining these methods with behavioral assays in future studies would be extremely helpful for better understanding the feeding behavior of native generalist insects and predicting their feeding responses to introduced host plants.

#### 3.2.2. Using Grasshopper Activity Measurements in Feeding Trials

Among the analyzed feeding records, trials that used plant damage and grasshopper consumption as measurements of grasshopper feeding preferences were significantly prevalent (86% and 90%, respectively). While measuring the grazed portion and amount of plant tissue consumed is often convenient in both the laboratory and field settings, the obtained data may not provide accurate information about food digestion, assimilation, and, ultimately, grasshopper growth and performance. Consumed plant species may not possess sufficient amount of nutrients (such as nitrogen) and, although a grasshopper can utilize such a plant, its growth and performance may not be positively affected compared with feeding on high nutritious plants. Meanwhile, maintaining high fitness while consuming plant tissues is an important characteristic of an insect candidate for effective biotic resistance. Plant damage and consumption measurements may also not be informative when they are applied to clipped plant material. Insect feeding preferences may be confounded by changes in plant secondary chemistry after clipping [27]. Consequently, an intact plant, which is unsuitable for grasshoppers, can become easily accessible if offered as clipped plant material. Thus, future studies that focus on insect preferences on native versus introduced host plants might want to use various measurements of grasshopper activity, specifically food assimilation, grasshopper body growth and performance. It would also be helpful to use intact plants (potted or in natural communities) in a combination with clipped plant material to account for possible differences in plant chemistry.

#### 3.2.3. Using Standardized Measurements 

Studies used in this systematic review greatly differed in the measurements used for assessing grasshopper host plant preferences. A total of 35 different measurements of feeding preferences, primarily associated with grasshopper consumption and development, were identified (Table S3). Few measurements of grasshopper host plant preferences were standardized in some way: Only 12 measurements were standardized by time, and of these, four measurements of food consumption were standardized by grasshopper weight. Although the meta-analysis showed that using measurements of actual consumption or relative estimates of food consumed (percentage, ranking, etc.) do not affect the outcomes of the feeding trials, it would be helpful for future studies on grasshopper preferences for host plants to use standardized measurements whenever it is possible. Such standardized units may not only be instrumental for meta-analyses of grasshopper food consumption on different plants, but may also help researchers apply similar experimental designs for multiple study species, and thus, may help facilitate a ‘transition’ for researchers between different study species if needed. 

### 3.3. Future Directions

Overall, this review has demonstrated the need for studies on the feeding response of acridid grasshoppers to their novel host plants. Such studies would be especially helpful to differentiate between mechanisms underlying the establishment of novel plant-insect associations. Particularly, this can help predict the result of interactions between native insects and introduced plants, such as ecological fitting, host shift, or an evolutionary trap when an insect readily accepts a novel plant on which it experiences low fitness [55]. Below are several specific research directions suggested for future studies on interactions between grasshoppers and their host plants, as well as general studies on the biotic resistance of native communities.

#### 3.3.1. Acridid Grasshoppers as a Study Object in Plant Invasion Ecology

This review has demonstrated that acridid grasshoppers are largely overlooked in studies in plant invasion ecology. Meanwhile, grasshopper species can be invaluable for our understanding of novel associations between introduced plants and native insect herbivores. Besides being a convenient study object, acridid grasshoppers play an important role in natural communities due to their abundance in different climatic regions of North America where they are one of the dominant generalist insect herbivores. In California’s grasslands, for example, grasshoppers are one of the dominant species among native generalist insect herbivores: There are 200 grasshopper species, with more than 50% being endemic [56,57]. 

#### 3.3.2. Combined Effect of the Plant Origin and Other Factors on Grasshopper Feeding Choice

Although this review focuses exclusively on the effect of the plant origin on grasshopper feeding choice, it has been demonstrated in previous studies that several other factors could potentially explain the attractiveness of introduced plants for native insects, such as grasshoppers. The most explored factors are the following: (a) Phylogenetic closeness of exotic plants to native plants [58]; (b) abundance of exotic plants in the introduced range [59]; and (c) perenniality of exotic plants [57]. Little is known, however, about how these factors interact with each other and which factor, or their combination, have the largest effect on grasshopper feeding choice. Litt et al. [43] pointed out that the relatedness of native and invasive plants might also help to explain variation in insect responses. It would be very helpful for future studies on biotic resistance to integrate these factors and further explore the potential role of native insects in preventing plant invasions.

#### 3.3.3. Time Since Introduction and Plant Resistant Traits

The longer insects are exposed to novel plants, the more likely they will be to use them as hosts [60]. However, even though native insects can use exotic plants as hosts, they might not be adapted yet to these novel hosts and, consequently, might not survive on them [43,60]. Thus, the time since introduction might be an important factor affecting the degree of establishment of novel plant-insect associations and the ability of native insects to suppress introduced, potentially invasive, plant populations. In addition, plant resistant traits might change over time since the time of introduction; this was demonstrated, for example, for *Solanum* plants [61]. Exploring herbivore resistant traits in the most preferred introduced plants was beyond the scope of this review, but it might be a focus of future studies. Also, since it is virtually impossible to assess the effect of each of the invasive plants on a native community, following Stout and Tiedeken [62], it is recommended that an analysis of individual-level traits on a case-by-case basis is conducted.

## 4. Materials and Methods

### 4.1. Literature Search

Following the PRISMA (Preferred Reporting Items for Systematic Reviews and Meta-Analyses) guidelines [63], six databases were searched for relevant studies on grasshopper feeding on native and introduced plants: JSTOR (1967–2017; accessed on 6-October-2017), ScienceDirect (1967–2017; accessed on 27-September-2017), SpringerLink (1967–2017; accessed on 26-September-2017), Web of Science (Biological Abstracts, 1995–2017; accessed on 28-September-2017), IngentaConnect (1998–2017; accessed on 27-September-2017), and Agricola (1974–2017; accessed on 25-September-2017). Additionally, the Journal of Orthoptera Research (2001–2016; accessed on 27-September-2017) was searched for relevant studies on grasshopper feeding. For the article search in each database, except Agricola, the following search terms were used: “Acrididae” AND “feeding” AND “plant”. For the Agricola database, a keyword, “grasshopper”, instead of “Acrididae” was used; and studies experimented with non-acridid grasshoppers were then manually excluded from subsequent article screening. The search results were then refined by language (“English”) and document type (“Article” and “Research reports”) where such refining options were available (SpringerLink, Web of Science, and JSTOR). Since a focus of this review is on experimental studies, document types such as books, book chapters, and reviews were excluded from subsequent analysis. The titles of the retrieved published studies (*n* = 2146) were screened, and studies with relevant titles (*n* = 411), i.e., containing words, such as “feeding”, “grasshoppers”, “host plants”, or “insect herbivores”, or their combination, were selected for further screening, which was conducted in two steps. First, the abstracts, and, in some cases, the main text of a paper, were screened for (1) grasshopper species origin, (2) study sites, (3) number of plants used, and (4) type of experiment (Step 1). Only studies that used North-American acridid grasshoppers and more than one plant species (offered simultaneously or consecutively), and that conducted behavioral feeding assays on the North-American continent (*n* = 87) were selected for the final screening. Studies that used crop content analysis, isotopic analysis, or molecular diet confirmation were excluded. The final screening (Step 2) was conducted using five inclusion criteria (described below).

### 4.2. Inclusion Criteria and Data Extraction

To be included in the analysis, a study had to fulfill all of the following criteria:

1. Use at least one plant that is native to North America and one plant that is exotic to North America (could be collected within or outside of North America);
2. Report grasshopper preference data for either each plant species or for a group of native plants versus exotic plants (studies reporting grasshopper feeding on plant mixtures versus a single diet, as well as on field sites dominated by a certain plant species, were excluded);
3. Report grasshopper preference for plant species growing at the same environmental conditions (studies comparing feeding, for example, at different elevations or temperatures were excluded);
4. Report grasshopper preference rather than acceptance of different plants; and
5. Use “direct” grasshopper feeding trials on different plant species without previous conditional feeding on a certain plant.

For the purpose of this review, only records of feeding preference trials were extracted, and a plant was considered to be a host plant if the record reported either (a) grasshopper feeding followed by measurement of grasshopper growth or performance; or (b) grasshopper feeding alone. Also, due to the focus of this review exclusively on the utilization or avoidance of introduced plants by grasshoppers, the authors’ rationale for choosing the study species was not considered. Plant native status (native or introduced), if not reported in a study, was determined using the USDA PLANT database. If the plant native status was different for Canada and the US, for the purpose of this review, the status reported for the region where the study was conducted was used. The origin and occurrence of grasshopper species (if not reported in a study) was determined using the Global Biodiversity Information Facility. After manual screening of the relevant studies (*n* = 87, from Step 1), 11 articles were found to satisfy all the inclusion criteria. In addition to 11 articles extracted from six databases and JOR, one relevant article was manually retrieved from ResearchGate, and one was obtained from another researcher (both satisfied the inclusion criteria). Each of the selected articles described one or more grasshopper feeding preference trials. Consequently, 13 articles containing a total of 63 records of separate feeding preference trials for 28 North-American grasshopper species were used for the analysis. 

To examine the feeding preferences of grasshoppers for native and introduced plants, for each record (i.e., feeding preference trial), the following data were extracted: Grasshopper species, native and introduced plant species, experimental conditions (environment, type of experiment), grasshopper life stage, measurements of grasshopper feeding preferences, and observed preference for plant species (most preferred, second preferred, and least preferred plants) (Table S1). 

To examine the invasive potential of the preferred introduced host plants of grasshoppers, for each most preferred and second preferred introduced plant species the following data were collected: (a) Plant family, duration, and growth habit (obtained from the USDA PLANT database); (b) U.S. Invasive Species Impact Rank (I-Rank), ecological impact (Subrank-I), current distribution and abundance (Subrank- II), trend in distribution/abundance (Subrank- III), and management difficulty (Subrank-IV) (obtained from the NatureServe database: http://explorer.natureserve.org/impact_rank.htm; the subranks address four major aspects of an invasive species’ total impact); (c) number of states where a plant is reported as invasive, and number of U.S. national parks where a plant is reported as invasive (obtained from the Center for Invasive Species and Ecosystem Health database and the Invasive Plant Atlas, respectively); and (d) grasshopper species reported to use a plant, and their host plant preference (Table S2).

### 4.3. Data Sources

The following resources (described above) were used to extract the data on grasshopper feeding preferences, species native and invasive status (both plant and grasshopper species), as well as the invasive potential of introduced plants:

- JSTOR: https://www.jstor.org/
- ScienceDirect: https://www.sciencedirect.com/
- SpringerLink: https://link.springer.com/
- Web of Science: https://clarivate.com/products/web-of-science/
- IngentaConnect: https://www.ingentaconnect.com/
- Agricola: https://agricola.nal.usda.gov/
- Journal of Orthoptera Research: https://jor.pensoft.net/
- The USDA PLANT database: https://plants.usda.gov/java/
- The Global Biodiversity Information Facility: https://www.gbif.org/
- The NatureServe database: http://www.natureserve.org/
- Center for Invasive Species and Ecosystem Health database: https://www.bugwood.org/
- The Invasive Plant Atlas: https://www.invasiveplantatlas.org/

The terms, “introduced”, “exotic”, and “invasive”, were used in this review following the definitions from Maryland.gov (Department of Natural resources: http://dnr.maryland.gov/Invasives/Pages/terminology.aspx).

### 4.4. Data Synthesis and Analysis

#### 4.4.1. Data Synthesis

The reported data on grasshopper feeding preferences under different experimental conditions, as well as for different grasshopper life stages, were first synthesized by using counts and proportions. Then, the prevalence of studies that reported introduced plants as the most preferred host plants of acridid grasshoppers was determined using a binomial test. The binomial test was also used to estimate the prevalence of studies using a certain type of experimental environment, plant material, preference trial, grasshopper life stage, general preference measurements, and grasshopper activity measurements. For the purpose of this review and to provide further methodological recommendations, the null hypothesis for the binomial test was that all the types of experimental conditions were presented in equal proportions in the published studies. Consequently, population proportions for each parameter were as follows: 0.25 (for experimental environment and grasshopper activity measurements), 0.5 (for plant material, preference trials, and preference outcomes), and 0.33 (for grasshopper life stage and general preference measurements).

For each parameter (see Table 1, column 1), the significance level was adjusted using false discovery rate correction [64]. For each feeding trial, from one to five different measurements were reported (Table S1), and a total of 35 different measurements of feeding preferences were identified (Table S3). For the purpose of this review, all the reported measurements were combined into four categories: (1) Percentage of leaf damage (e.g. percent of leaf area removed); (2) preference ratings (e.g. the intensity of leaf feeding); (3) plant biomass consumed (g or cm3); and (4) grasshopper food assimilation, performance, and development (e.g. body mass, number of fecal pellets, consumption rate, etc.). 

#### 4.4.2. Meta-analysis

To estimate the effect of the experimental environment, plant material, grasshopper life stages, and measurement units used in feeding trials, a preference metric (PrM) was derived from each feeding record:

PrM = (n _*most preferred introduced plant species*_ − n _*most preferred native plant species*_)/n _*total plant species offered*_, (3)

Next, the standard error (SE), variance (Var), and a separate metric of weighted effect size (W * es) were derived from the preference metric [38]:

W * es = PrM * W,(4)

where W is the weight of each feeding record [38]:

W = 1/(SE)^2^ (5)

Following Hedge et al. [65], this preference metric (PrM) was chosen as a meaningful summary of each feeding record. This modified response ratio quantified the proportion of introduced plants consumed by grasshoppers compared to the corresponding proportion of consumed native plants.

For a feeding record to be included in this analysis, it had to provide information about the most preferred introduced and native plant species identified during the feeding trial. Thus, the records with “no-preference” outcomes (8 out of 63 records) were excluded from this analysis (Table S4).

Following closely the steps also described in Neyeloff et al. [38], the Q test and I^2^ method were performed to quantify the heterogeneity among studies and to decide on the effect summary model (Table S4). Based on the Q and I^2^ values, a random effects model was chosen to meta-analyze the data extracted from the feeding records [38]. Following Neyeloff et al. [38], all the calculations, as well as building the forest plot, were performed in Excel. 

The Kruskal–Wallis test (due to a lack of normality of the data) followed by the post hoc Nemenyi test was conducted to test whether effect sizes differed among the feeding trials with various experimental conditions. These tests were conducted in R, v.3.4.3 [66]. 

## 5. Conclusions

In conclusion, it is very encouraging that the number of studies on grasshopper preferences has increased during the last two decades; this corresponds with the general increase of studies on invasion biology [67]. Even though the retrieved studies greatly differed in their experimental design and measurements used to quantify grasshopper host plant preferences, the lack of grasshopper avoidance of novel host plants was consistently observed across studies. Furthermore, this review has shown that by demonstrating a preference for introduced, often highly invasive, plants, acridid grasshoppers possess a high potential to contribute to the biotic resistance of native communities to plant invasions. This can be further explored in future experimental studies; insights from this review can also be helpful for developing effective restoration programs. 

## Figures and Tables

**Table 1:** Experimental conditions and outcomes extracted from the feeding records.

Parameter	Type	Number of Records	% of Total	*p*-Value, Binomial Test	(*i*/*m*) * Q
experimental environment	common garden	19	30	*p* = 0.382	0.0375
	greenhouse	4	6	*p* < 0.001 *	0.0125
	laboratory (clipped plant leaves)	9	14	*p* = 0.057	0.0025
	laboratory (clipped plant stems with leaves)	31	49	*p* < 0.001 *	0.0125
plant material	intact plants	23	37	*p* = 0.04	0.025
	clipped plants	40	63	*p* = 0.04	0.025
preference trial	choice (two plant species)	2	3	*p* < 0.001 *	0.016
	choice (plant mixture)	55	87	*p* < 0.001 *	0.016
	no-choice	6	10	*p* < 0.001 *	0.016
grasshopper life stage	adults	37	59	*p* < 0.001 *	0.016
	nymphs	20	32	*p* = 0.894	0.033
	mixed	6	10	*p* < 0.001 *	0.016
general preference measurements	plant damage	54	86	*p* < 0.001 *	0.016
	grasshopper activity	4	6	*p* < 0.001 *	0.016
	mixed	5	8	*p* < 0.001 *	0.016
grasshopper activity measurements	growth and performance	1	2	*p* < 0.001 *	0.0125
	consumption	57	90	*p* < 0.001 *	0.0125
	assimilation	1	2	*p* < 0.001 *	0.0125
	mixed	4	6	*p* < 0.001 *	0.0125
grasshopper most preferred host plants	native plants	11	17	*p* < 0.001 *	0.0125
	introduced plants	43	68	*p* = 0.005 *	0.025
	mixed	7	11	*p* < 0.001 *	0.0125
	no preferences	2	3	*p* < 0.001 *	0.0125
grasshopper least preferred host plants	native plants	28	44	*p* = 0.45	0.025
	introduced plants	5	8	*p* < 0.001 *	0.008
	mixed	13	21	*p* < 0.001 *	0.008
	no preferences	6	10	*p* < 0.001 *	0.008
	not reported	11	17	*p* < 0.001 *	0.008

*: *p* values with asterisks (‘‘*’’) are significant at the corresponding Benjamini-Hochberg critical values [*(i*/*m*) * Q; where Q = 0.05, *i* is a *p*-value rank, and *m* is the total number of different parameter types used].

## Supplementary Materials

The following are available online at http://www.mdpi.com/2223-7747/7/4/83/s1, Table S1: Experimental settings and feeding preferences of acridid grasshoppers reported in extracted studies, Table S2: Introduced plants which are preferred by acridid grasshoppers, Table S3: Measurements of grasshopper preferences for host plants reported in the studies, Table S4: Intermediate calculations of the effect size needed for the meta-analysis. Random-effects model, Table S5: Data for the forest plot, Figure S1: Literature search and data collection: PRISMA flowchart (modified from Moher et al. [63]), Figure S2: Number of studies on feeding of acridid grasshoppers published during 1967- 2017: (a) all studies retrieved from the SpringerLink database; (b) studies retrieved from six databases and included in the analysis.

## References

[1] Pimentel D., Zuniga R., Morrison D. (2005). Update on the environmental and economic costs associated with alien-invasive species in the United States. Ecol. Econ..

[2] Liebhold A.M., Brockerhoff E.G., Garrett L.J., Parke J.L., Britton K.O. (2012). Live plant imports: the major pathway for forest insect and pathogen invasions of the US. Front. Ecol. Environ..

[3] Alpert P. (2006). The advantages and disadvantages of being introduced. Biol. Invas..

[4] Pyšek P., Jarošík V., Pergl J. (2011). Alien plants introduced by different pathways differ in invasion success: unintentional introductions as a threat to natural areas. PLoS ONE.

[5] Pimentel D., McNair S., Janecka J., Wightman J., Simmonds C., O’connell C., Wong E., Russel L., Zern J., Aquino T. (2001). Economic and environmental threats of alien plant, animal, and microbe invasions. Agric. Ecosyst. Environ..

[6] Catford J.A., Jansson R., Nilsson C. (2009). Reducing redundancy in invasion ecology by integrating hypotheses into a single theoretical framework. Divers. Distrib..

[7] Keane R.M., Crawley M.J. (2002). Exotic plant invasions and the enemy release hypothesis. Trends Ecol. Evol..

[8] Blossey B., Notzold R. (1995). Evolution of increased competitive ability in invasive nonindigenous plants: A hypothesis. J. Ecol..

[9] Callaway R.M., Ridenour W.M. (2004). Novel weapons: Invasive success and the evolution of increased competitive ability. Front. Ecol. Environ..

[10] Müller-Schärer H., Schaffner U., Steinger T. (2004). Evolution in invasive plants: implications for biological control. Trends Ecol. Evol..

[11] Siemann E., Rogers W.E. (2003). Reduced resistance of invasive varieties of the alien tree *Sapium sebiferum* to a generalist herbivore. Oecologia.

[12] Harmoney K.R., Hickman K.R. (2004). Comparative morphology of Caucasian old world bluestem and native grasses. Agron. J..

[13] Jogesh T., Carpenter D., Cappuccino N. (2008). Herbivory on invasive exotic plants and their non-invasive relatives. Biol. Invas..

[14] Schmidt C.D., Hickman K.R., Channell R., Harmoney K., Stark W. (2008). Competitive abilities of native grasses and non-native (*Bothriochloa* spp.) grasses. Plant Ecol..

[15] Beaton L.L., Van Zandt P.A., Esselman E.J., Knight T.M. (2011). Comparison of the herbivore defense and competitive ability of ancestral and modern genotypes of an invasive plant, *Lespedeza cuneata*. Oikos.

[16] Elton C.S. (2000). The Ecology of Invasions by Animals and Plants.

[17] Simberloff D., Von Holle B. (1999). Positive interactions of nonindigenous species: Invasional meltdown?. Biol. Invas..

[18] Maron J.L., Vilà M. (2001). When do herbivores affect plant invasion? Evidence for the natural enemies and biotic resistance hypotheses. Oikos.

[19] Parker J.D., Hay M.E. (2005). Biotic resistance to plant invasions? Native herbivores prefer non-native plants. Ecol. Lett..

[20] Levine J.M., Adler P.B., Yelenik S.G. (2004). A meta-analysis of biotic resistance to exotic plant invasions. Ecol. Lett..

[21] Agrawal A.A., Kotanen P.M. (2003). Herbivores and the success of exotic plants: a phylogenetically controlled experiment. Ecol. Lett..

[22] Parker J.D., Burkepile D.E., Hay M.E. (2006). Opposing effects of native and exotic herbivores on plant invasions. Science.

[23] Hull-Sanders H.M., Clare R., Johnson R.H., Meyer G.A. (2007). Evaluation of the evolution of increased competitive ability (EICA) hypothesis: Loss of defense against generalist but not specialist herbivores. J. Chem. Ecol..

[24] Zou J., Siemann E., Rogers W.E., DeWalt S.J. (2008). Decreased resistance and increased tolerance to native herbivores of the invasive plant Sapium sebiferum. Ecography.

[25] Fielding D.J., Conn J.S. (2011). Feeding preference for and impact on an invasive weed (*Crepis tectorum*) by a native, generalist insect herbivore, *Melanoplus borealis* (Orthoptera: Acrididae). Ann. Entomol. Soc. Am..

[26] Fan S., Yu D., Liu C. (2013). The invasive plant *Alternanthera philoxeroides* was suppressed more intensively than its native congener by a native generalist: implications for the biotic resistance hypothesis. PLoS ONE.

[27] Avanesyan A., Culley T.M. (2015). Herbivory of native and exotic North-American prairie grasses by nymph *Melanoplus* grasshoppers. Plant Ecol..

[28] Avanesyan A., Culley T.M. (2015). Feeding preferences of *Melanoplus femurrubrum* grasshoppers on native and exotic grasses: behavioral and molecular approaches. Entomol. Exp. Appl..

[29] Morrison W.E., Hay M.E. (2011). Herbivore preference for native *vs*. exotic plants: Generalist herbivores from multiple continents prefer exotic plants that are evolutionarily naïve. PLoS ONE.

[30] Capinera J.L., Scott R.D., Walker T.J. (2004). Field Guide to Grasshoppers, Crickets, and Katydids of the United States.

[31] Saul W.C., Jeschke J.M. (2015). Eco-evolutionary experience in novel species interactions. Ecol. Lett..

[32] Avanesyan A., Culley T.M. (2017). Tolerance of native and exotic prairie grasses to herbivory by *Melanoplus* grasshoppers: Application of a nondestructive method for estimating plant biomass changes as a response to herbivory. J. Torr. Bot. Soc..

[33] Cumberland C., Jonas J.L., Paschke M.W. (2017). Impact of grasshoppers and an invasive grass on establishment and initial growth of restoration plant species. Restoration Ecol..

[34] Schaffner U., Ridenour W.M., Wolf V.C., Bassett T., Müller C., Müller-Schärer H., Sutherland S., Lortie C.J., Callaway R.M. (2011). Plant invasions, generalist herbivores, and novel defense weapons. Ecology.

[35] Jonas J.L., Joern A. (2013). Dietary selection and nutritional regulation in a common mixed-feeding insect herbivore. Entomol. Exp. Appl..

[36] Lankau R.A., Rogers W.E., Siemann E. (2004). Constraints on the utilisation of the invasive Chinese tallow tree *Sapium sebiferum* by generalist native herbivores in coastal prairies. Ecol. Entomol..

[37] Whipple S.D., Brust M.L., Hoback W.W., Farnsworth-Hoback K.M. (2009). The grasshoppers *Arphia xanthoptera* and *Dichromorpha viridis* prefer introduced smooth brome over other grasses. Great Plains Res..

[38] Neyeloff J.L., Fuchs S.C., Moreira L.B. (2012). Meta-analyses and Forest plots using a Microsoft excel spreadsheet: Step-by-step guide focusing on descriptive data analysis. BMC Res. Notes.

[39] Begna S.H., Fielding D.J. (2003). Damage potential of grasshoppers (Orthoptera: Acrididae) on early growth stages of small-grains and canola under subarctic conditions. J. Econ. Entomol..

[40] Joern A., Behmer S.T. (1998). Impact of diet quality on demographic attributes in adult grasshoppers and the nitrogen limitation hypothesis. Ecol. Entomol..

[41] Belovsky G.E., Slade J.B. (2018). Grasshoppers affect grassland ecosystem functioning: Spatial and temporal variation. Basic Appl. Ecol..

[42] Otte D. (1975). Plant preference and plant succession: A consideration of evolution and plant preference in Schistocerca. Oecologia.

[43] Litt A.R., Cord E.E., Fulbright T.E., Schuster G.L. (2014). Effects of invasive plants on arthropods. Conserv. Biol..

[44] Bezemer T.M., Harvey J.A., Cronin J.T. (2014). Response of native insect communities to invasive plants. Annu. Rev. Entomol..

[45] Dillemuth F.P., Rietschier E.A., Cronin J.T. (2009). Patch dynamics of a native grass in relation to the spread of invasive smooth brome (*Bromus inermis*). Biol. Invas..

[46] Chu I.W., Knutson H. (1970). Preferences of eight grasshopper among eleven species of cultivated grasses. J. Kans. Entomol. Soc..

[47] Hewitt G.B., Blickenstaff C.C. (1974). Evaluation of methods for screening grasses for resistance to grasshopper feeding. J. Range Manage..

[48] Branson D.H., Sword G.A. (2009). Grasshopper herbivory affects native plant diversity and abundance in a grassland dominated by the exotic grass *Agropyron cristatum*. Restoration Ecol..

[49] Jones C.G., Coleman J.S. (1988). Leaf disc size and insect feeding preference: implications for assays and studies on induction of plant defense. Entomol. Exp. Appl..

[50] Peterson C.H., Renaud P.E. (1989). Analysis of feeding preference experiments. Oecologia.

[51] Avanesyan A. (2014). Plant DNA detection from grasshopper guts: A step-by-step protocol, from tissue preparation to obtaining plant DNA sequences. Appl. Plant Sci..

[52] McClenaghan B., Gibson J.F., Shokralla S., Hajibabaei M. (2015). Discrimination of grasshopper (Orthoptera: Acrididae) diet and niche overlap using next-generation sequencing of gut contents. Ecol. Evol..

[53] Huang X., Wu H., McNeill M.R., Qin X., Ma J., Tu X., Cao G., Wang G., Nong X., Zhang Z. (2016). Quantitative analysis of diet structure by real-time PCR, reveals different feeding patterns by two dominant grasshopper species. Sci. Rep..

[54] Huang X., McNeill M.R., Ma J., Qin X., Tu X., Cao G., Wang G., Nong X., Zhang Z. (2017). Gut transcriptome analysis shows different food utilization efficiency by the grasshopper *Oedaleous asiaticus* (Orthoptera: Acrididae). J. Econ. Entomol..

[55] Sunny A., Diwakar S., Sharma G.P. (2015). Native insects and invasive plants encounters. Arthropod-Plant Int..

[56] Joern A. (1989). Insect herbivory in the transition to California annual grasslands: Did grasshoppers deliver the coup de grass?. Grassland Structure and Function.

[57] Stromberg M.R., Corbin J.D., D’Antonio C.M. (2007). California grasslands: ecology and management. California grassland restoration. Ecology and Management of California Grasslands.

[58] Lambdon P.W., Hulme P.E. (2006). How strongly do interactions with closely-related native species influence plant invasions? Darwin’s naturalization hypothesis assessed on Mediterranean islands. J. Biogeography.

[59] Boys H.A. (1978). Food selection by *Oedaleus senegalensis* (Acrididae: Orthoptera) in grassland and millet fields. Entomol. Exp. Appl..

[60] deJonge R.B., Bourchier R.S., Smith S.M. (2017). Initial response by a native beetle, *Chrysochus auratus* (Coleoptera: Chrysomelidae), to a novel introduced host-plant, *Vincetoxicum rossicum* (Gentianales: Apocynaceae). Environ. Entomol..

[61] Flanders K.L., Hawkes J.G., Radcliffe E.B., Lauer F.I. (1992). Insect resistance in potatoes: Sources, evolutionary relationships, morphological and chemical defenses, and ecogeographical associations. Euphytica.

[62] Stout J.C., Tiedeken E.J. (2017). Direct interactions between invasive plants and native pollinators: Evidence, impacts and approaches. Funct. Ecol..

[63] Moher D., Liberati A., Tetzlaff J., Altman D.G. (2009). Prisma Group. Preferred reporting items for systematic reviews and meta-analyses: the PRISMA statement. PLoS Medicine.

[64] Benjamini Y., Hochberg Y. (1995). Controlling the false discovery rate: A practical and powerful approach to multiple testing. J. Royal Stat. Soc. Series B.

[65] Hedges L.V., Gurevitch J., Curtis P.S. (1999). The meta-analysis of response ratios in experimental ecology. Ecology.

[66] R Core Team (2013) (2014). R: A language and environment for statistical computing.

[67] Richardson D.M., Pyšek P. (2008). Fifty years of invasion ecology–the legacy of Charles Elton. Divers. Distrib..

