# Supplementary material for: Should I Eat or Should I Go? Acridid Grasshoppers and Their Novel Host Plants: Potential for Biotic Resistance
Source: Plants (Basel). 2018 Oct 7;7(4):83. doi: 10.3390/plants7040083 (PMC6313845; doi:10.3390/plants7040083)
Supplement: Supplementary file 1 [file plants-07-00083-s001.zip › SM_revised2/FigS2.pdf]

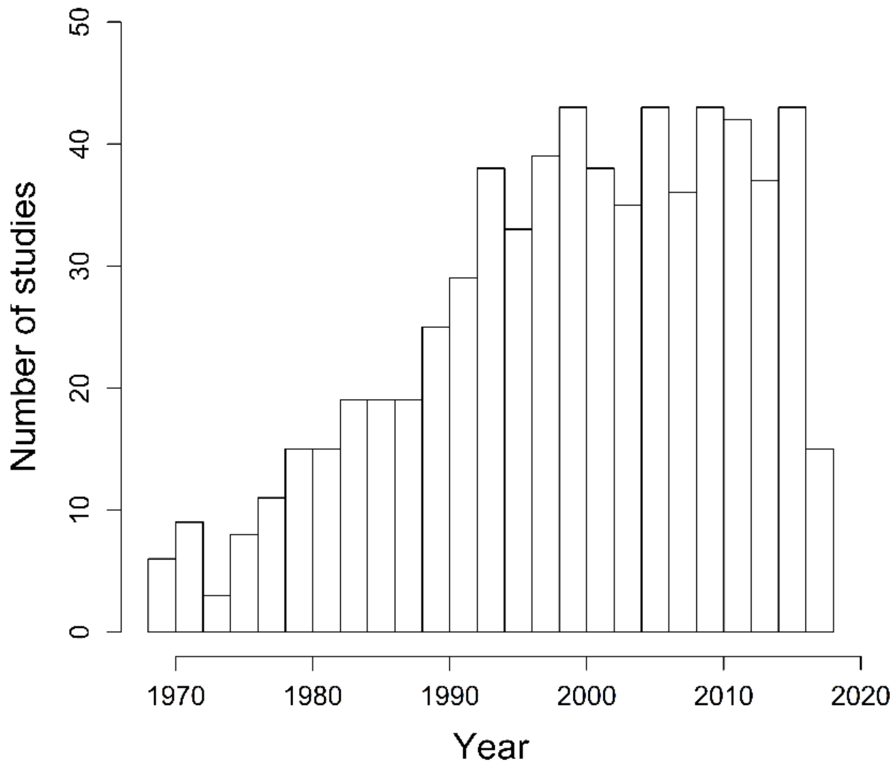

(a)

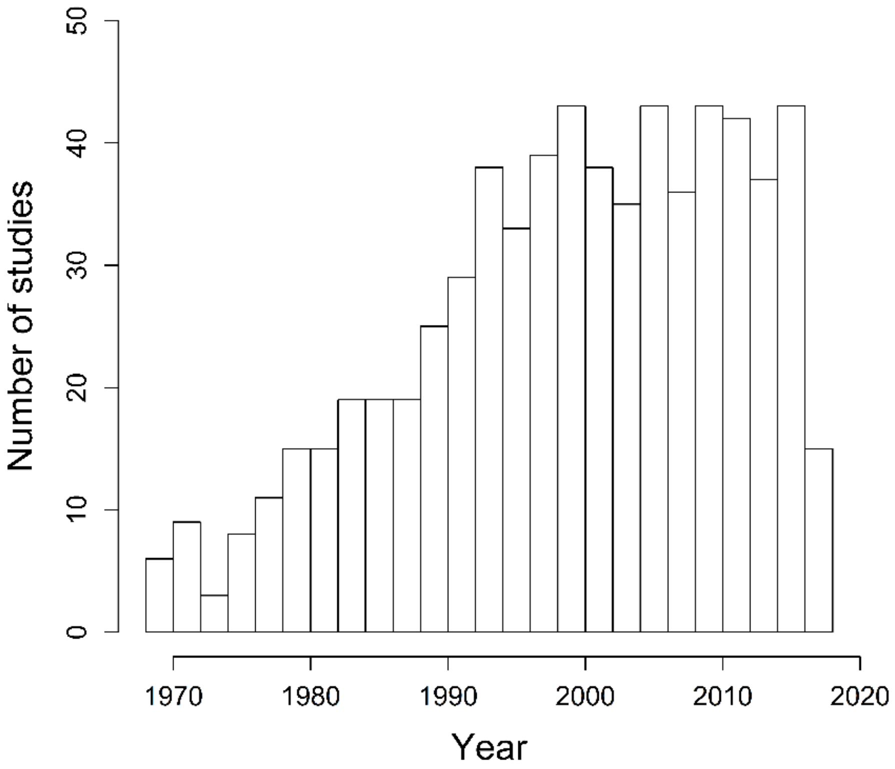

(b)

Figure S2. Number of studies on feeding of acridid grasshoppers published during 1967- 2017: **(a)** all studies retrieved from the SpringerLink database; **(b)** studies retrieved from six databases and included in the analysis.
