# Supplementary material for: Should I Eat or Should I Go? Acridid Grasshoppers and Their Novel Host Plants: Potential for Biotic Resistance
Source: Plants (Basel). 2018 Oct 7;7(4):83. doi: 10.3390/plants7040083 (PMC6313845; doi:10.3390/plants7040083)
Supplement: Supplementary file 1 [file plants-07-00083-s001.zip › SM_revised2/TableS1.docx]

**Table S1. Experimental settings and feeding preferences of acridid grasshoppers reported in extracted studies.**

| # | Grasshopper species | Introduced plants | Native plants | Experimental settings | | Grasshopper life stage | Measurements | Preferences reported | | | Author, year |
| --- | --- | --- | --- | --- | --- | --- | --- | --- | --- | --- | --- |
|  |  |  |  | Environment, plant material | Type of experiment |  |  | Most preferred plant | Second preferred plant | Least preferred plant |  |
| 1 | *Ageneotettix deorum* | *Agropyron trichophorum*  *Elymus junceus*  *Dactylis glomerata*  *Bromus inermis* | *Stipa viridula*  *Agropyron dasystachyum* | Common garden | Choice | Nymph | Relative percentage eaten  seedling plants | *Dactylis glomerata*  *Bromus*  *inermis* | N/A | *Stipa viridula*  *Elymus junceus* | Hewitt and Blickenstaff  1974 |
| 2 | *Ageneotettix deorum* | *Agropyron trichophorum*  *Elymus junceus*  *Dactylis glomerata*  *Bromus inermis* | *Stipa viridula*  *Agropyron dasystachyum* | Common garden | Choice | Nymph | Relative percentage eaten  advanced plants | *Dactylis glomerata*  *Bromus*  *inermis* | N/A | *Stipa viridula*  *Elymus junceus* | Hewitt and Blickenstaff  1974 |
| 3 | *Ageneotettix deorum* | *Agropyron trichophorum*  *Elymus junceus*  *Dactylis glomerata*  *Bromus inermis* | *Stipa viridula*  *Agropyron dasystachyum* | Common garden | Choice | Adult | Relative percentage eaten  seedling plants | *Dactylis glomerata*  *Bromus*  *inermis* | N/A | *Stipa viridula*  *Elymus junceus* | Hewitt and Blickenstaff  1974 |
| 4 | *Ageneotettix deorum* | *Agropyron trichophorum*  *Elymus junceus*  *Dactylis glomerata*  *Bromus inermis* | *Stipa viridula*  *Agropyron dasystachyum* | Common garden | Choice | adult | Relative percentage eaten  advanced plants | *Dactylis glomerata*  *Bromus*  *inermis* | N/A | *Stipa viridula*  *Elymus junceus* | Hewitt and Blickenstaff  1974 |
| 5 | *Ageneotettex deorum deorum* | *Thinopyrum ponticum Bromus inermis**  *Dactylis glomerata Psathyrostachys juncea*  *Schedonorus arundinaceus*  *Phleum pratense* | *Pascopyrum smithii Andropogon gerardi*  *Bouteloua*  *curtipendula*  *Phalaris arundinacea Panicum virgatum* | Lab (cuttings) | choice | adult | The intensity of leaf feeding  The intensity of inflorescence and seed feeding | *Andropogon gerardi*  *Pascopyrum smithii*  *Schedonorus arundinaceus*  *Bromus inermis* | N/A  N/A | *Phalaris arundinacea*  *Panicum virgatum* | Chu and Knutson 1970 |
| 6 | *Aptenopedes sphenariodes* | *Paspalum notatum*  *Fluegge * - both* | *Elephantopus elatus*  *Erechtites*  *hieraciifolia*  *Pityopsis graminifolia*  *Fuirena squarrosa* | Lab (clipped cuttings) | choice | Adult | mean consumption | *Elephantopus elatus* | *Erechtites hieraciifolia* | *P. notatum.* | Smith and Capinera  2005 |
| 7 | *Arphia xanthoptera* | *Bromus inermis*  *Poa pratensis* | *Andropogon gerardii*  *Bouteloua curtipendula* | Lab (plant cuttings 25 cm) | choice | adult | plant biomass consumed | *Bromus inermis*  *Poa pratensis* | N/A | *Bouteloua, Andropogon* | Whipple et al 2009 |
| 8 | *Bruneria brunnea* | *Agropyron trichophorum*  *Elymus junceus*  *Dactylis glomerata*  *Bromus inermis* | *Stipa viridula*  *Agropyron dasystachyum* | Common garden | Choice | Adult | Relative percentage eaten  seedling plants | *Dactylis glomerata*  *Bromus*  *inermis* | N/A | *Stipa viridula*  *Elymus junceus* | Hewitt and Blickenstaff  1974 |
| 9 | *Bruneria brunnea* | *Agropyron trichophorum*  *Elymus junceus*  *Dactylis glomerata*  *Bromus inermis* | *Stipa viridula*  *Agropyron dasystachyum* | Common garden | Choice | Adult | Relative percentage eaten  advanced plants | *Dactylis glomerata*  *Bromus*  *inermis* | N/A | *Stipa viridula*  *Elymus junceus* | Hewitt and Blickenstaff  1974 |
| 10 | *Camnula pellucida* | *Agropyron trichophorum*  *Elymus junceus*  *Dactylis glomerata*  *Bromus inermis* | *Stipa viridula*  *Agropyron dasystachyum* | Common garden | Choice | nymph | Relative percentage eaten  seedling plants | *Dactylis glomerata*  *Bromus*  *inermis* | N/A | *Stipa viridula*  *Elymus junceus* | Hewitt and Blickenstaff  1974 |
| 11 | *Camnula pellucida* | *Agropyron trichophorum*  *Elymus junceus*  *Dactylis glomerata*  *Bromus inermis* | *Stipa viridula*  *Agropyron dasystachyum* | Common garden | Choice | nymph | Relative percentage eaten  advanced plants | *Dactylis glomerata*  *Bromus*  *inermis* | N/A | *Stipa viridula*  *Elymus junceus* | Hewitt and Blickenstaff  1974 |
| 12 | *Camnula pellucida* | *Agropyron trichophorum*  *Elymus junceus*  *Dactylis glomerata*  *Bromus inermis* | *Stipa viridula*  *Agropyron dasystachyum* | Common garden | Choice | Adult | Relative percentage eaten  seedling plants | *Dactylis glomerata*  *Bromus*  *inermis* | N/A | *Stipa viridula*  *Elymus junceus* | Hewitt and Blickenstaff  1974 |
| 13 | *Camnula pellucida* | *Agropyron trichophorum*  *Elymus junceus*  *Dactylis glomerata*  *Bromus inermis* | *Stipa viridula*  *Agropyron dasystachyum* | Common garden | Choice | adult | Relative percentage eaten  advanced plants | *Dactylis glomerata*  *Bromus*  *inermis* | N/A | *Stipa viridula*  *Elymus junceus* | Hewitt and Blickenstaff  1974 |
| 14 | *Chortophaga australior* | *Eleusine indica*  *Paspalum notatum*  *Fluegge * - both* | *Digitaria ciliaris*  *Amaranthus spinosus*  *Cenchrus echinatus* | Lab (clipped cuttings) | choice | adult | mean consumption | *Eleusine indica* | *Digitaria ciliaris* | *Cenchrus echinatu* | Smith and Capinera  2005 |
| 15 | *Dichromorpha viridis* | *Bromus inermis*  *Poa pratensis* | *Andropogon gerardii*  *Bouteloua curtipendula* | Lab (plant cuttings 25 cm) | choice | Adult | plant biomass consumed | *Bromus inermis*  *Poa pratensis* | N/A | N/A | Whipple et al 2009 |
| 16 | *Eritettix obscurus* | *Paspalum notatum*  *Fluegge * - both* | *Aristida beyrichiana* | Lab (clipped cuttings) | choice | Adult | mean consumption | *Aristida beyrichiana* | *Paspalum notatum* | N/A | Smith and Capinera  2005 |
| 17 | *Gymnoscirtetes pusillus* | *Eichhornia crassipes*  *Urochloa mutica*  *Leptochloa spp.**  *Panicum repens*  *Hydrocotyle spp.**  *Sesbania herbacea ** | *Typha spp.*  *Pontederia cordata*  *Sacciolepis striata*  *Chasmanthium sessiliflorum*  *Polygonum punctatum*  *Polygonum hirsutum*  *Cicuta maculata*  *Ludwigia octovalvis*  *Ludwigia suffruticosa*  *Sagittaria latifolia*  *Cyperus compressus*  *Juncus effusus* | Lab (cuttings) | choice | Nymphs and adults  (mix) | mean consumption value | *Typha spp.*  *Ludwigia octovalvis*  *Ludwigia suffruticosa*  *Leptochloa spp.* | N/A | N/A | Squitier and Capinera 2002 |
| 18 | *Leptysma marginicollis* | *Eichhornia crassipes*  *Urochloa mutica*  *Leptochloa spp.**  *Panicum repens*  *Hydrocotyle spp.**  *Sesbania herbacea ** | *Typha spp.*  *Pontederia cordata*  *Sacciolepis striata*  *Chasmanthium sessiliflorum*  *Polygonum punctatum*  *Polygonum hirsutum*  *Cicuta maculata*  *Ludwigia octovalvis*  *Ludwigia suffruticosa*  *Sagittaria latifolia*  *Cyperus compressus*  *Juncus effusus* | Lab (cuttings) | choice | Nymphs and adults  (mix) | mean consumption value | *Typha spp.*  *Pontederia cordata*  *Ludwigia octovalvis*  *Juncus effusus* | N/A | N/A | Squitier and Capinera 2002 |
| 19 | *Melanoplus angustipennis* | *Sapium sebiferum* | *Celtis laevigata*  *Liquidambar styracifula*  *Platanus occidentalis* | Lab (leaves) | choice | adult | the amount of biomass consumed  the per cent of total leaf area removed | *Sapium*  *no differences* | *Celtis*  *no differences* | *Liquidambar Platanus*  *no differences* | Lankau et al. 2004 |
| 20 | *Melanoplus bispinosus* | *Brassica kaber*  *Paspalum notatum*  *Fluegge * - both* | *Richardia scabra*  *Gnaphalium pensylvanicum*  *Gnaphalium pensylvanicum*  *Digitaria bicornis* | Lab (clipped cuttings) | choice | adult | mean consumption | *Richardia scabra* | *Paspalum notatum* | *Digitaria bicornis* | Smith and Capinera  2005 |
| 21 | *Melanoplus bivittatus* | *Thinopyrum ponticum Bromus inermis**  *Dactylis glomerata Psathyrostachys juncea*  *Schedonorus arundinaceus*  *Phleum pratense* | *Pascopyrum smithii Andropogon gerardi*  *Bouteloua*  *curtipendula*  *Phalaris arundinacea Panicum virgatum* | Lab (cuttings) | choice | nymphs | The intensity of leaf feeding  The intensity of inflorescence and seed feeding | *Dactylis glomerata*  *Bromus inermis*  *Psathyrostachys juncea* | *Phleum pratense*  N/A | *Thinopyrum ponticum*  *Bouteloua*  *Curtipendula*  *Thinopyrum ponticum* | Chu and Knutson 1970 |
| 22 | *Melanoplus bivittatus* | *Thinopyrum ponticum Bromus inermis**  *Dactylis glomerata Psathyrostachys juncea*  *Schedonorus arundinaceus*  *Phleum pratense* | *Pascopyrum smithii Andropogon gerardi*  *Bouteloua*  *curtipendula*  *Phalaris arundinacea Panicum virgatum* | Lab (cuttings) | choice | adults | The intensity of leaf feeding  The intensity of inflorescence and seed feeding | *Bromus inermis*  *Schedonorus arundinaceus* | *Schedonorus arundinaceus*  N/A | *Andropogon gerardi*  *Bouteloua curtipendula*  *Bouteloua*  *curtipendula* | Chu and Knutson 1970 |
| 23 | *Melanoplus bivittatus* | *Bromus tectorum* | *17 native plant species* | Greenhouse (leaves) | paired choice^**^ | 2-5^th^ instars,  adults (mix) | plant biomass consumed | *Bromus tectorum* | N/A | N/A | Cumberland et al 2017 |
| 24 | *Melanoplus borealis* | *Crepis tectorum*  *Taraxacum officinale*  *Bromus inermis** | *Dracocephalum parviflorum* | Greenhouse | choice | 5^th^ instar | proportions of the total plant weight in a pot | *Taraxacum officinale* | *Bromus inermis* | *Dracocephalum parviflorum* | Fielding and Conn 2011 |
| 25 | *Melanoplus borealis* | *Crepis tectorum*  *Taraxacum officinale*  *Bromus inermis* | *Dracocephalum parviflorum*  *Chamerion angustifolium* | Lab (leaves) | paired choice | adult | Leaf area consumed (transformed to dry weight) | *Taraxacum officinale* | *Crepis tectorum* | *Dracocephalum, Chamerion* | Fielding and Conn 2011 |
| 26 | *Melanoplus differentialis* | *Thinopyrum ponticum Bromus inermis**  *Dactylis glomerata Psathyrostachys juncea*  *Schedonorus arundinaceus*  *Phleum pratense* | *Pascopyrum smithii Andropogon gerardi*  *Bouteloua*  *curtipendula*  *Phalaris arundinacea Panicum virgatum* | Lab (cuttings) | choice | nymphs | The intensity of leaf feeding  The intensity of inflorescence and seed feeding | *Bromus inermis*  *Psathyrostachys juncea*  *Dactylis glomerata*  *Phleum pratense*  *Psathyrostachys juncea*  *Schedonorus arundinaceus* | N/A  N/A | *Thinopyrum ponticum*  *Bouteloua*  *curtipendula*  *Thinopyrum ponticum*  *Phleum pratense* | Chu and Knutson 1970 |
| 27 | *Melanoplus differentialis* | *Thinopyrum ponticum Bromus inermis**  *Dactylis glomerata Psathyrostachys juncea*  *Schedonorus arundinaceus*  *Phleum pratense* | *Pascopyrum smithii Andropogon gerardi*  *Bouteloua*  *curtipendula*  *Phalaris arundinacea Panicum virgatum* | Lab (cuttings) | choice | adults | The intensity of leaf feeding  The intensity of inflorescence and seed feeding | *Bromus inermis*  *Schedonorus arundinaceus*  *Schedonorus arundinaceus* | N/A  N/A | *Bouteloua*  *curtipendula*  N/A | Chu and Knutson 1970 |
| 28 | *Melanoplus differentialis* | *Cynodon dactylon Zoysia matrella*  *Festuca arundinacea*  *Sorghum halepense* | *Buchloe dactyloids*  *Stenotaphrum secundatum*  *Zoysia japonica*  *Poa arachnifera* | Lab (leaves) | no-choice | adult | amount of feeding (rating)  number of fecal pellets over 8-day period  weight of fecal pellets over 8-day period | *Festuca arundinacea*  *Festuca arundinacea*  *Festuca arundinacea* | *Poa pratensis x P. arachnifera*    N/A  N/A | *Zoysia matrella*  N/A  N/A | Reinert et al 2011 |
| 29 | *Melanoplus differentialis* | *Nerium oleander*  *Campsis grandiflora Schum*  *Canna x generalis*  *Ipomoea batatas*  *Lagerstroemia fauriei*  *Bougainvillea spp.*  *Comm. ex Juss*  *Cortaderia selloana*  *Sorghum halepense*  *Plumbago auriculata*  *Petunia violacea*  *Glandularia hybrida*  *Rosa sp. ** | *Tecoma stans*  *Hibiscus moscheutos*  *Phlox paniculata*  *Lantana horrida* | Lab (leaves) | no-choice | adult | amount of feeding (rating)  number of fecal pellets in the first 3d  weight of fecal pellets in the first 3d | *Plumbago auriculata*  *Glandularia hybrida*  *Canna x *generalis*  *Sorghum halepense*  *Cortaderia selloana*    *Canna x *generalis*  *Glandularia hybrida*  *Canna x *generalis*  *Glandularia hybrida* | N/A  N/A  N/A | *Hibiscus moscheutos*  *Petunia violacea*  *Phlox paniculata*  *Tecoma stans*  *Campsis grandiflora*  *Ipomoea batatas*  *Bougainvillea spp.*  *Lantana horrida*  *Ipomoea batatas*  *Bougainvillea spp.*  *Lantana horrida* | Reinert et al 2011 |
| 30 | *Melanoplus femurrubrum* | *Miscanthus sinensis*  *Bothriochloa ischaemum* | *Andropogon gerardii*  *Bouteloua curtipendula* | Lab (leaves) | choice | 3 & 4^th^ instars | The total leaf biomass consumed  the proportion of the amount of leaf tissue consumed | none  none | none  none | none  none | Avanesyan and Culley 2015b |
| 31 | *Melanoplus femurrubrum* | *Miscanthus sinensis*  *Bothriochloa ischaemum* | *Andropogon gerardii*  *Bouteloua curtipendula* | Common garden | choice | 3 & 4^th^ instars | total volume of the grazed portion  number of missing tips per plant | *Miscanthus sinensis*  Exotics > natives  Exotics > natives | none | none | Avanesyan and Culley 2015b |
| 32 | *Melanoplus femurrubrum* | *Miscanthus sinensis*  *Bothriochloa ischaemum* | *Andropogon gerardii*  *Bouteloua curtipendula* | Greenhouse | choice | 3 & 4^th^ instars | total volume of the grazed portion  number of missing tips per plant | *Miscanthus sinensis*  Exotics > natives  Exotics > natives | none  none | none  none | Avanesyan and Culley 2015b |
| 33 | *Melanoplus femurrubrum* | *Miscanthus sinensis*  *Bothriochloa ischaemum* | *Andropogon gerardii*  *Bouteloua curtipendula* | Greenhouse | no-choice | 3 & 4^th^ instars | total volume of the grazed portion  number of missing tips per plant  grasshopper body mass  grasshopper body length | no differences  no differences  no differences  Exotics > natives | N/A  N/A  N/A  N/A | N/A  N/A  N/A  N/A | Avanesyan and Culley 2015b |
| 34 | *Melanoplus femurrubrum* | *Thinopyrum ponticum Bromus inermis**  *Dactylis glomerata Psathyrostachys juncea*  *Schedonorus arundinaceus*  *Phleum pratense* | *Pascopyrum smithii Andropogon gerardi*  *Bouteloua*  *curtipendula*  *Phalaris arundinacea Panicum virgatum* | Lab (cuttings) | choice | nymphs | The intensity of leaf feeding  The intensity of inflorescence and seed feeding | *Phleum pratense*  *Bromus inermis*  *Psathyrostachys juncea* | N/A  N/A | *Panicum virgatum*  *Bouteloua*  *curtipendula*  *Thinopyrum ponticum* | Chu and Knutson 1970 |
| 35 | *Melanoplus femurrubrum* | *Thinopyrum ponticum Bromus inermis**  *Dactylis glomerata Psathyrostachys juncea*  *Schedonorus arundinaceus*  *Phleum pratense* | *Pascopyrum smithii Andropogon gerardi*  *Bouteloua*  *curtipendula*  *Phalaris arundinacea Panicum virgatum* | Lab (cuttings) | choice | adults | The intensity of leaf feeding  The intensity of inflorescence and seed feeding | *Bromus inermis*  *Schedonorus arundinaceus*  *Schedonorus arundinaceus* | N/A  N/A | *Bouteloua*  *curtipendula*  *Bouteloua*  *curtipendula* | Chu and Knutson 1970 |
| 36 | *Melanoplus femurrubrum* | *Miscanthus sinensis*  *Bothriochloa ischaemum* | *Andropogon gerardii*  *Bouteloua curtipendula* | Lab (leaves) | choice | adult | relative consumption rate  fresh-weight consumption index | No differences  No differences | None  None | None  None | Avanesyan and Culley 2015a |
| 37 | *Melanoplus femurrubrum* | *Miscanthus sinensis*  *Bothriochloa ischaemum* | *Andropogon gerardii*  *Bouteloua curtipendula* | Lab (leaves) | no-choice | adult | relative assimilation rate  approximate digestibility | no differences  Natives > exotics | None  None | None  None | Avanesyan and Culley 2015a |
| 38 | *Melanoplus femurrubrum* | *Miscanthus sinensis*  *Bothriochloa ischaemum* | *Andropogon gerardii*  *Bouteloua curtipendula* | Greenhouse | choice | adult | total volume of the grazed portion  feeding rate | Exotics > natives  no differences | None  none | None  none | Avanesyan and Culley 2015a |
| 39 | *Melanoplus femurrubrum* | *Miscanthus sinensis*  *Bothriochloa ischaemum* | *Andropogon gerardii*  *Bouteloua curtipendula* | Common garden | choice | adult | total volume of the grazed portion  feeding rate | Exotics > natives  Exotics > natives | None  none | *Bouteloua curtipendula*  *Bouteloua curtipendula* | Avanesyan and Culley 2015a |
| 40 | *Melanoplus keeleri luridus* | *Thinopyrum ponticum Bromus inermis**  *Dactylis glomerata Psathyrostachys juncea*  *Schedonorus arundinaceus*  *Phleum pratense* | *Pascopyrum smithii Andropogon gerardi*  *Bouteloua*  *curtipendula*  *Phalaris arundinacea Panicum virgatum* | Lab (cuttings) | choice | nymphs | The intensity of leaf feeding  The intensity of inflorescence and seed feeding | *Schedonorus arundinaceus*  *Schedonorus arundinaceus* | *Bromus inermis*  N/A | *Bouteloua*  *curtipendula*  *Phalaris arundinacea*  *Bromus inermis* | Chu and Knutson 1970 |
| 41 | *Melanoplus querneus* | *Paspalum notatum*  *Fluegge * - both* | *Habenaria floribunda*  *Smilax bona-nox*  *Quercus nigra*  *Clematis reticulata*  *Aster dumosus* | Lab (clipped cuttings) | choice | adult | mean consumption | *Habenaria floribunda* | *Smilax bona-nox* | *Aster dumosus* | Smith and Capinera  2005 |
| 42 | *Melanoplus sanguinipes* | *Bromus inermis**  *Agropyron cristatum Thinopyrum intermedium*  *Thinopyrum ponticum*  *Leymus angustus*  *Psathyrostachys juncea*  *Bromus biebersteinii* | *Agropyron trachycaulum*  *Pascopyrum smithii* | Common garden | No-choice | 2^nd^ instar | Mean dry weight of grasshoppers  Development  mortality | *Pascopyrum smithii*  *Pascopyrum smithii*  *Thinopyrum intermedium*  No differences | N/A  N/A  No differences | *Bromus inermis*  *Bromus inermis*  No differences | Olfert et al 1994 |
| 43 | *Melanoplus sanguinipes* | *Lolium multiflorum* | *Bouteloua curtipendula* | Lab (leaves) | no-choice | 4^th^ instar | Final dry weights of grasshoppers  growth rate  consumption rate  approximate digestibility  efficiency of conversion of ingested material  efficiency of conversion of digested material | *Lolium*  *Lolium*  *no differences*  *Lolium*  *Lolium*  *no differences* | N/A  N/A  N/A  N/A | *Bouteloua*  *Bouteloua*  *Bouteloua*  *Bouteloua* | Barbehenn et al 2004 |
| 44 | *Melanoplus sanguinipes* | *Agropyron trichophorum*  *Elymus junceus*  *Dactylis glomerata*  *Bromus*  *inermis* | *Stipa viridula*  *Agropyron dasystachyum* | Common garden | Choice | Nymph | Relative percentage eaten  seedling plants | *Dactylis glomerata*  *Bromus*  *inermis* | N/A | *Stipa viridula*  *Elymus junceus* | Hewitt and Blickenstaff  1974 |
| 45 | *Melanoplus sanguinipes* | *Agropyron trichophorum*  *Elymus junceus*  *Dactylis glomerata*  *Bromus*  *inermis* | *Stipa viridula*  *Agropyron dasystachyum* | Common garden | Choice | Nymph | Relative percentage eaten  advanced plants | *Dactylis glomerata*  *Bromus*  *inermis* | N/A | *Stipa viridula*  *Elymus junceus* | Hewitt and Blickenstaff  1974 |
| 46 | *Melanoplus sanguinipes* | *Thinopyrum ponticum Bromus inermis**  *Dactylis glomerata Psathyrostachys juncea*  *Schedonorus arundinaceus*  *Phleum pratense* | *Pascopyrum smithii Andropogon gerardi*  *Bouteloua*  *curtipendula*  *Phalaris arundinacea Panicum virgatum* | Lab (cuttings) | choice | nymphs | The intensity of leaf feeding  The intensity of inflorescence and seed feeding | *Bromus inermis*  *Dactylis glomerata*  *Phleum pratense*  *Schedonorus arundinaceus*  *Psathyrostachys juncea* | N/A  N/A | *Bouteloua*  *curtipendula*  *Bouteloua*  *curtipendula* | Chu and Knutson 1970 |
| 47 | *Melanoplus sanguinipes* | *Thinopyrum ponticum Bromus inermis**  *Dactylis glomerata Psathyrostachys juncea*  *Schedonorus arundinaceus*  *Phleum pratense* | *Pascopyrum smithii Andropogon gerardi*  *Bouteloua*  *curtipendula*  *Phalaris arundinacea Panicum virgatum* | Lab (cuttings) | choice | adults | The intensity of leaf feeding  The intensity of inflorescence and seed feeding | *Bromus inermis*  *Schedonorus arundinaceus*  *Psathyrostachys juncea* | N/A  N/A | *Bouteloua*  *curtipendula*  *Thinopyrum ponticum*  *Andropogon gerardi* | Chu and Knutson 1970 |
| 48 | *Melanoplus sanguinipes* | *Agropyron trichophorum*  *Elymus junceus*  *Dactylis glomerata*  *Bromus*  *inermis* | *Stipa viridula*  *Agropyron dasystachyum* | Common garden | Choice | Adult | Relative percentage eaten  seedling plants | *Dactylis glomerata*  *Bromus*  *inermis* | N/A | *Stipa viridula*  *Elymus junceus* | Hewitt and Blickenstaff  1974 |
| 49 | *Melanoplus sanguinipes* | *Agropyron trichophorum*  *Elymus junceus*  *Dactylis glomerata*  *Bromus*  *inermis* | *Stipa viridula*  *Agropyron dasystachyum* | Common garden | Choice | adult | Relative percentage eaten  advanced plants | *Dactylis glomerata*  *Bromus*  *inermis* | N/A | *Stipa viridula*  *Elymus junceus* | Hewitt and Blickenstaff  1974 |
| 50 | *Metator pardalinus* | *Agropyron trichophorum*  *Elymus junceus*  *Dactylis glomerata*  *Bromus*  *inermis* | *Stipa viridula*  *Agropyron dasystachyum* | Common garden | Choice | adult | Relative percentage eaten  seedling plants | *Dactylis glomerata*  *Bromus*  *inermis* | N/A | *Stipa viridula*  *Elymus junceus* | Hewitt and Blickenstaff  1974 |
| 51 | *Metator pardalinus* | *Agropyron trichophorum*  *Elymus junceus*  *Dactylis glomerata*  *Bromus*  *inermis* | *Stipa viridula*  *Agropyron dasystachyum* | Common garden | Choice | adult | Relative percentage eaten  advanced plants | *Dactylis glomerata*  *Bromus*  *inermis* | N/A | *Stipa viridula*  *Elymus junceus* | Hewitt and Blickenstaff  1974 |
| 52 | *Orphulella speciosa* | *Thinopyrum ponticum Bromus inermis**  *Dactylis glomerata Psathyrostachys juncea*  *Schedonorus arundinaceus*  *Phleum pratense* | *Pascopyrum smithii Andropogon gerardi*  *Bouteloua*  *curtipendula*  *Phalaris arundinacea Panicum virgatum* | Lab (cuttings) | choice | nymphs | The intensity of leaf feeding  The intensity of inflorescence and seed feeding | *Dactylis glomerata*  *Psathyrostachys juncea* | N/A  N/A | *N/A*  *Thinopyrum ponticum* | Chu and Knutson 1970 |
| 53 | *Orphulella speciosa* | *Thinopyrum ponticum Bromus inermis**  *Dactylis glomerata Psathyrostachys juncea*  *Schedonorus arundinaceus*  *Phleum pratense* | *Pascopyrum smithii Andropogon gerardi*  *Bouteloua*  *curtipendula*  *Phalaris arundinacea Panicum virgatum* | Lab (cuttings) | choice | adults | The intensity of leaf feeding  The intensity of inflorescence and seed feeding | *Andropogon gerardi*  *Schedonorus arundinaceus* | *Bromus inermis*  *Schedonorus arundinaceus*  *Psathyrostachys juncea* | *Phalaris arundinacea*  *Thinopyrum ponticum* | Chu and Knutson 1970 |
| 54 | *Paroxya atlantica* | *Eichhornia crassipes*  *Urochloa mutica*  *Leptochloa spp.**  *Panicum repens*  *Hydrocotyle spp.**  *Sesbania herbacea ** | *Typha spp.*  *Pontederia cordata*  *Sacciolepis striata*  *Chasmanthium sessiliflorum*  *Polygonum punctatum*  *Polygonum hirsutum*  *Cicuta maculata*  *Ludwigia octovalvis*  *Ludwigia suffruticosa*  *Sagittaria latifolia*  *Cyperus compressus*  *Juncus effusus* | Lab (cuttings) | choice | adult | mean consumption value | *Typha spp.*  *Hydrocotyle spp.*  *Sagittaria latifolia*  *Cyperus compressus*  *Cicuta maculata* | N/A | N/A | Squitier and Capinera 2002 |
| 55 | *Paroxya clavuliger* | *Paspalum notatum*  *Fluegge * - both* | *Fuirena squarrosa,*  *Sagittaria lancifolia,*  *Sesbania exaltata*  *Limnobium spongia* | Lab (clipped cuttings) | choice | adult | mean consumption | *Limnobium spongia* | *Fuirena squarrosa* | *Paspalum notatum* | Smith and Capinera  2005 |
| 56 | *Paroxya clavuliger* | *Eichhornia crassipes*  *Urochloa mutica*  *Leptochloa spp.**  *Panicum repens*  *Hydrocotyle spp.**  *Sesbania herbacea ** | *Typha spp.*  *Pontederia cordata*  *Sacciolepis striata*  *Chasmanthium sessiliflorum*  *Polygonum punctatum*  *Polygonum hirsutum*  *Cicuta maculata*  *Ludwigia octovalvis*  *Ludwigia suffruticosa*  *Sagittaria latifolia*  *Cyperus compressus*  *Juncus effusus* | Lab (cuttings) | choice | Nymphs and adults  (mix) | mean consumption value | *Typha spp.*  *Eichhornia crassipes*  *Polygonum punctatum*  *Hydrocotyle spp.*  *Cicuta maculata*  *Leptochloa spp.* | N/A | N/A | Squitier and Capinera 2002 |
| 57 | *Phoetaliotes nebrascensis* | *Thinopyrum ponticum Bromus inermis**  *Dactylis glomerata Psathyrostachys juncea*  *Schedonorus arundinaceus*  *Phleum pratense* | *Pascopyrum smithii Andropogon gerardi*  *Bouteloua*  *curtipendula*  *Phalaris arundinacea Panicum virgatum* | Lab (cuttings) | choice | nymphs | The intensity of leaf feeding  The intensity of inflorescence and seed feeding | *Bromus inermis*  *Schedonorus arundinaceus*  *Andropogon gerardi*  *Schedonorus arundinaceus*  *Panicum virgatum*  *Schedonorus arundinaceus* | N/A  N/A | N/A  *Phalaris arundinacea* | Chu and Knutson 1970 |
| 58 | *Phoetaliotes nebrascensis* | *Thinopyrum ponticum Bromus inermis**  *Dactylis glomerata Psathyrostachys juncea*  *Schedonorus arundinaceus*  *Phleum pratense* | *Pascopyrum smithii Andropogon gerardi*  *Bouteloua*  *curtipendula*  *Phalaris arundinacea Panicum virgatum* | Lab (cuttings) | choice | adults | The intensity of leaf feeding  The intensity of inflorescence and seed feeding | *Bromus inermis*  *Schedonorus arundinaceus* | *Andropogon gerardi*  N/A | *Bouteloua curtipendula*  *Andropogon gerardi* | Chu and Knutson 1970 |
| 59 | *Romalea microptera* | *Paspalum notatum*  *Fluegge * - both* | *Sagittaria lancifolia*  *Habenaria floribunda*  *Smilax bona-nox*  *Quercus nigra*  *Aster dumosus* | Lab (clipped cuttings) | choice | adult | mean consumption | *Sagittaria lancifolia* | *Habenaria floribunda* | *Aster dumosus* | Smith and Capinera  2005 |
| 60 | *Romalea microptera* | *Eichhornia crassipes*  *Urochloa mutica*  *Leptochloa spp.**  *Panicum repens*  *Hydrocotyle spp.**  *Sesbania herbacea ** | *Typha spp.*  *Pontederia cordata*  *Sacciolepis striata*  *Chasmanthium sessiliflorum*  *Polygonum punctatum*  *Polygonum hirsutum*  *Cicuta maculata*  *Ludwigia octovalvis*  *Ludwigia suffruticosa*  *Sagittaria latifolia*  *Cyperus compressus*  *Juncus effusus* | Lab (cuttings) | choice | Nymphs and adults  (mix) | mean consumption value | *Typha spp.*  *Sagittaria latifolia*  *Cyperus compressus*  *Ludwigia suffruticosa*  *Leptochloa*  *spp.*  *Polygonum hirsutum* | N/A | N/A | Squitier and Capinera 2002 |
| 61 | *Schistocerca amerincana* | *Digitaria ischemum*  *Cyperus esculentus**  *Panicum maximum*  *Paspalum notatum*  *Fluegge * - both* | *Chamaesyce*  *hyssopifolia*  *Sesbania exaltata*  *Smilax bona-nox*  *Cyperus echinatus*  *Chamaecrista fasciculate*  *Panicum hemitomon* | Lab (clipped cuttings) | choice | adult | mean consumption | *Digitaria ischemum Chamaesyce hyssopifolia,* | *Sesbania exaltata* | *Paspalum notatum* | Smith and Capinera  2005 |
| 62 | *Schistocerca ceratiola* | *Paspalum notatum*  *Fluegge * - both* | *Ceratiola ericoides* | Lab (clipped cuttings) | choice | adult | mean consumption | *Ceratiola ericoides* | N/A | N/A | Smith and Capinera  2005 |
| 63 | *Stenacris vitreipennis* | *Eichhornia crassipes*  *Urochloa mutica*  *Leptochloa spp.**  *Panicum repens*  *Hydrocotyle spp.**  *Sesbania herbacea ** | *Typha spp.*  *Pontederia cordata*  *Sacciolepis striata*  *Chasmanthium sessiliflorum*  *Polygonum punctatum*  *Polygonum hirsutum*  *Cicuta maculata*  *Ludwigia octovalvis*  *Ludwigia suffruticosa*  *Sagittaria latifolia*  *Cyperus compressus*  *Juncus effusus* | Lab (cuttings) | choice | Nymphs and adults  (mix) | mean consumption value | *Typha spp.*  *Cyperus compressus* | N/A | N/A | Squitier and Capinera 2002 |

*** plant species which has both introduced and native status

**** a paired choice represents a trial with 2 plant species only (1 native and 1 introduced), while a choice experiment uses a mixture of plants (more than 2 species)

References:

1. Avanesyan A, Culley TM (2015a) Herbivory of native and exotic North-American prairie grasses by nymph Melanoplus grasshoppers. Plant Ecol 216:451-464
2. Avanesyan A, Culley TM (2015b) Feeding preferences of *Melanoplus femurrubrum* grasshoppers on native and exotic grasses: behavioral and molecular approaches. Entomol Exp Appl 157:152-163.
3. Barbehenn RV, Karowe DN, Chen Z (2004) Performance of a generalist grasshopper on a C_3_ and a C_4_ grass: compensation for the effects of elevated CO_2_ on plant nutritional quality. Oecologia 140:96-103
4. Chu IW, Knutson H (1970) Preferences of eight grasshopper among eleven species of cultivated grasses. J Kans Entomol Soc 43:20-31.
5. Cumberland C, Jonas JL, Paschke MW (2017) Impact of grasshoppers and an invasive grass on establishment and initial growth of restoration plant species. Restoration Ecol 25:385-395
6. Fielding DJ, Conn JS (2011) Feeding preference for and impact on an invasive weed (Crepis tectorum) by a native, generalist insect herbivore, Melanoplus borealis (Orthoptera: Acrididae). Ann Entomol Soc Am 104:1303-1308
7. Hewitt GB, Blickenstaff CC (1974) Evaluation of methods for screening grasses for resistance to grasshopper feeding. J Range Manage 27:285-287
8. Lankau RA, Rogers WE, Siemann E (2004) Constraints on the utilisation of the invasive Chinese tallow tree Sapium sebiferum by generalist native herbivores in coastal prairies. Ecol Entomol 29:66-75
9. Olfert O, Hinks CF, Weiss RM, Wright SB (1994) The effect of perennial grasses on growth, development and survival of grasshopper nymphs (Orthoptera: Acrididae): Implications for population management in roadsides. J Orthoptera Res 2:1-3
10. Smith TR, Capinera JL (2005) Host preferences and habitat associations of some Florida grasshoppers (Orthoptera: Acrididae). Environ Entomol 34:210-224
11. Squitier JM, Capinera JL (2002) Host selection by grasshoppers (Orthoptera: Acrididae) inhabiting semi-aquatic environments. Florida Entomol 85:336-340
12. Reinert JA, Mackay W, Engelke MC, George SW (2011) The differential grasshopper (Orthoptera: Acrididae)—Its impact on turfgrass and landscape plants in urban environs. Florida Entomol 94:253-261
13. Whipple SD, Brust ML, Hoback WW, Farnsworth-Hoback KM (2009) The grasshoppers Arphia xanthoptera and Dichromorpha viridis prefer introduced smooth brome over other grasses. Great Plains Res 19:179-186
