# Supplementary material for: Should I Eat or Should I Go? Acridid Grasshoppers and Their Novel Host Plants: Potential for Biotic Resistance
Source: Plants (Basel). 2018 Oct 7;7(4):83. doi: 10.3390/plants7040083 (PMC6313845; doi:10.3390/plants7040083)
Supplement: Supplementary file 1 [file plants-07-00083-s001.zip › SM_revised2/TableS2.docx]

**Table S2. Introduced plants which are preferred by acridid grasshoppers**

| Plant species | Family  Duration  Growth habit | I-Rank  Year of review^1^ | Ecol. Impact | Current Distrib. and Abund. | Trend in Distrib./  Abund. | Manag. Difficulty | #U.S. states where reported invasive | #U.S. National Parks where reported invasive | Grasshopper species | Grasshopper host plant preference | Author, year |
| --- | --- | --- | --- | --- | --- | --- | --- | --- | --- | --- | --- |
| *Dactylis glomerata* L*.*  orchardgrass | Poaceae Perennial Graminoid | M/I  2005 | L/I | M | M/L | M/L | 1 | 10 | *Ageneotettix deorum*  *Bruneria brunnea*  *Camnula pellucida*  *Melanoplus sanguinipes*  *Metator pardalinus*  *Melanoplus bivittatus*  *Melanoplus differentialis*  *Orphulella speciose* | Most preferred plant | Hewitt and Blickenstaff  1974  Chu and Knutson 1970 |
| *Bromus inermis* Leyss. **  smooth brome | Poaceae Perennial Graminoid | H/M  2007 | M | H | M/L | M | 3 | 8 | *Ageneotettix deorum*  *Bruneria brunnea*  *Camnula pellucida*  *Melanoplus sanguinipes*  *Metator pardalinus*  *Ageneotettix deorum*  *Arphia xanthoptera*  *Dichromorpha viridis*  *Melanoplus bivittatus*  *Melanoplus differentialis*  *Melanoplus femurrubrum*  *Phoetaliotes nebrascensis*  *Melanoplus borealis*  *Orphulella speciosa* | Most and second preferred plant | Hewitt and Blickenstaff  1974  Chu and Knutson 1970  Whipple et al 2009  Fielding and Conn 2011 |
| *Bromus tectorum* L.  cheatgrass | Poaceae Annual Graminoid | H  2004 | H | H/M | M | H/M | 6 | 16 | *Melanoplus bivittatus* | Most preferred plant | Cumberland et al 2017 |
| *Canna ×generalis* L.H. Bailey (pro sp.) [glauca × indica]  canna lily | Cannaceae Perennial Forb/herb | NYA | NYA | NYA | NYA | NYA | NA | NA | *Melanoplus differentialis* | Most preferred plant | Reinert et al 2011 |
| *Cortaderia selloana* (Schult. & Schult. f.) Asch. & Graebn. Uruguayan pampas grass | Poaceae Perennial Graminoid | M/L  2004 | L | M | M | U | 2 | 2 | *Melanoplus differentialis* | Most preferred plant | Reinert et al 2011 |
| *Crepis tectorum* L.  narrowleaf hawksbeard | Asteraceae  Annual  Forb/herb | NYA | NYA | NYA | NYA | NYA | NA | 1 | *Melanoplus borealis* | Second preferred plant | Fielding and Conn 2011 |
| *Digitaria ischaemum* (Schreb.) Schreb. ex Muhl.  smooth crabgrass | Poaceae Annual Graminoid | NYA | NYA | NYA | NYA | NYA | NA | NA | *Schistocerca amerincana* | Most preferred plant | Smith and Capinera  2005 |
| *Eleusine indica* (L.) Gaertn. Indian goosegrass | Poaceae Annual Graminoid | NYA | NYA | NYA | NYA | NYA | 2 | 1 | *Chortophaga australior* | Most preferred plant | Smith and Capinera  2005 |
| *Eichhornia crassipes* (Mart.) Solms  common water hyacinth | Pontederiaceae Perennial Forb/herb | H  2004 | H/M | H | H/M | H | 9 | NA | *Paroxya clavuliger* | Most preferred plant | Squitier and Capinera 2002 |
| *Glandularia ×hybrida* (Groenl. & Rümpler) G.L. Nesom & Pruski [peruviana × phlogiflora or platensis]  mock vervain | Verbenaceae Perennial Forb/herb | NYA | NYA | NYA | NYA | NYA | NA | NA | *Melanoplus differentialis* | Most preferred plant | Reinert et al 2011 |
| *Lolium perenne* L. ssp. multiflorum (Lam.) Husnot  Italian ryegrass | Poaceae Annual  Perennial Graminoid | NA | NA | NA | NA | NA | 1 | 1 | *Melanoplus sanguinipes* | Most preferred plant | Barbehenn et al 2004 |
| *Miscanthus sinensis* Andersson  Chinese silvergrass | Poaceae Perennial Graminoid | M  2007 | M/L | H | M | M/L | 8 | 4 | *Melanoplus femurrubrum* | Most preferred plant | Avanesyan and Culley 2015b  Avanesyan and Culley 2015a |
| *Paspalum notatum* Flueggé bahiagrass * | Poaceae Perennial Graminoid | I  2004 | NA | NA | NA | NA | 2 | NA | *Eritettix obscurus* | Secondpreferred plant | Smith and Capinera  2005 |
| *Phleum pretense* L.  timothy | Poaceae Perennial Graminoid | M  2006 | M/L | H | M | M | 1 | 5 | *Melanoplus differentialis*  *Melanoplus femurrubrum*  *Melanoplus sanguinipes*  *Melanoplus bivittatus* | Most and second preferred plant | Chu and Knutson 1970 |
| *Plumbago auriculata* Lam.  Cape leadwort | Plumbaginaceae Perennial Forb/herb  Shrub  Vine | NYA | NYA | NYA | NYA | NYA | NA | NA | *Melanoplus differentialis* | Most preferred plant | Reinert et al 2011 |
| *Poa pratensis* L.  Kentucky bluegrass | Poaceae Perennial Graminoid | M  2005 | M | H | M/L | M/L | 3 | NA | *Arphia xanthoptera* | Most preferred plant | Whipple et al 2009 |
| *Psathyrostachys juncea* (Fisch.) Nevski  Russian wildrye | Poaceae Perennial Graminoid | L/I  2005 | L/I | M/L | L | M/I | NA | NA | *Melanoplus bivittatus*  *Melanoplus differentialis*  *Melanoplus femurrubrum*  *Melanoplus sanguinipes*  *Orphulella speciosa* | Most and second preferred plant | Chu and Knutson 1970 |
| *Triadica sebifera* (L.) Small Chinese tallow | Euphorbiaceae Perennial  Tree | H  2004 | H | M | H/M | H/M | 9 | 1 | *Melanoplus angustipennis* | Most preferred plant | Lankau et al. 2004 |
| *Schedonorus arundinaceus* (Schreb.) Dumort., nom. cons.  tall fescue | Poaceae Perennial Graminoid | H/M  2005 | M | H | M | H/M | 7 | NA | *Ageneotettix deorum*  *Melanoplus bivittatus*  *Melanoplus differentialis*  *Melanoplus femurrubrum*  *Melanoplus keeleri luridus*  *Melanoplus sanguinipes*  *Orphulella speciose*  *Phoetaliotes nebrascensis*  *Melanoplus bivittatus* | Most and second preferred plant | Chu and Knutson 1970  Reinert et al 2011 |
| *Sorghum halepense* (L.) Pers. Johnsongrass | Poaceae Perennial Graminoid | H/M  2006 | M/L | H | M/L | H/M | 25 | 18 | *Melanoplus differentialis* | Most preferred plant | Reinert et al 2011 |
| *Taraxacum officinale* F.H. Wigg. ssp. officinale  common dandelion | Asteraceae Perennial Forb/herb | NYA | NYA | NYA | NYA | NYA | NA | 7 | *Melanoplus borealis* | Most preferred plant | Fielding and Conn 2011 |
| *Thinopyrum intermedium* (Host) Barkworth & D.R. Dewey  intermediate wheatgrass | Poaceae Perennial Graminoid | M/I  2005 | L/I | M/L | H/L | M/L | NA | NA | *Melanoplus sanguinipes* | Most preferred plant | Olfert et al 1994 |

^1^ Invasive rank: High – H; Medium – M; Low – L; Insignificant – I; Unknown – U; Not yet assessed – NYA; Not available - NA

References:

1. Avanesyan A, Culley TM (2015a) Herbivory of native and exotic North-American prairie grasses by nymph Melanoplus grasshoppers. Plant Ecol 216:451-464
2. Avanesyan A, Culley TM (2015b) Feeding preferences of *Melanoplus femurrubrum* grasshoppers on native and exotic grasses: behavioral and molecular approaches. Entomol Exp Appl 157:152-163.
3. Barbehenn RV, Karowe DN, Chen Z (2004) Performance of a generalist grasshopper on a C_3_ and a C_4_ grass: compensation for the effects of elevated CO_2_ on plant nutritional quality. Oecologia 140:96-103
4. Chu IW, Knutson H (1970) Preferences of eight grasshopper among eleven species of cultivated grasses. J Kans Entomol Soc 43:20-31.
5. Cumberland C, Jonas JL, Paschke MW (2017) Impact of grasshoppers and an invasive grass on establishment and initial growth of restoration plant species. Restoration Ecol 25:385-395
6. Fielding DJ, Conn JS (2011) Feeding preference for and impact on an invasive weed (Crepis tectorum) by a native, generalist insect herbivore, Melanoplus borealis (Orthoptera: Acrididae). Ann Entomol Soc Am 104:1303-1308
7. Hewitt GB, Blickenstaff CC (1974) Evaluation of methods for screening grasses for resistance to grasshopper feeding. J Range Manage 27:285-287
8. Lankau RA, Rogers WE, Siemann E (2004) Constraints on the utilisation of the invasive Chinese tallow tree Sapium sebiferum by generalist native herbivores in coastal prairies. Ecol Entomol 29:66-75
9. Olfert O, Hinks CF, Weiss RM, Wright SB (1994) The effect of perennial grasses on growth, development and survival of grasshopper nymphs (Orthoptera: Acrididae): Implications for population management in roadsides. J Orthoptera Res 2:1-3
10. Smith TR, Capinera JL (2005) Host preferences and habitat associations of some Florida grasshoppers (Orthoptera: Acrididae). Environ Entomol 34:210-224
11. Squitier JM, Capinera JL (2002) Host selection by grasshoppers (Orthoptera: Acrididae) inhabiting semi-aquatic environments. Florida Entomol 85:336-340
12. Reinert JA, Mackay W, Engelke MC, George SW (2011) The differential grasshopper (Orthoptera: Acrididae)—Its impact on turfgrass and landscape plants in urban environs. Florida Entomol 94:253-261
13. Whipple SD, Brust ML, Hoback WW, Farnsworth-Hoback KM (2009) The grasshoppers Arphia xanthoptera and Dichromorpha viridis prefer introduced smooth brome over other grasses. Great Plains Res 19:179-186
