# Supplementary material for: Should I Eat or Should I Go? Acridid Grasshoppers and Their Novel Host Plants: Potential for Biotic Resistance
Source: Plants (Basel). 2018 Oct 7;7(4):83. doi: 10.3390/plants7040083 (PMC6313845; doi:10.3390/plants7040083)
Supplement: Supplementary file 1 [file plants-07-00083-s001.zip › SM_revised2/TableS3.docx]

**Table S3. Measurements of grasshopper preferences for host plants reported in the studies.**

| **#** | **Measurements** | **Units** |
| --- | --- | --- |
| 1 | Relative percentage eaten seedling plants | % |
| 2 | Relative percentage eaten advanced plants | % |
| 3 | The intensity of leaf feeding | rank |
| 4 | The intensity of inflorescence and seed | rank |
| 5 | Mean consumption | rank |
| 6 | Plant biomass consumed | g |
| 7 | Mean consumption value | rank |
| 8 | The amount of biomass consumed | g |
| 9 | The per cent of total leaf area removed | % |
| 10 | Proportions of the total plant weight in a pot | proportion |
| 11 | Leaf area consumed (transformed to dry weight) | g |
| 12 | Amount of feeding | rank |
| 13 | Number of fecal pellets over 8-day period | number |
| 14 | Weight of fecal pellets over 8-day period | g |
| 15 | Number of fecal pellets in the first 3d | number |
| 16 | Weight of fecal pellets in the first 3d | g |
| 17 | The total leaf biomass consumed | g |
| 18 | The proportion of the amount of leaf tissue consumed | proportion |
| 19 | Total volume of the grazed portion | cm^3^ number of grasshoppers^-1^ day^-1^ |
| 20 | Number of missing tips per plant | Number day^-1^ |
| 21 | Grasshopper body mass | g day^-1^ |
| 22 | Grasshopper body length | cm day^-1^ |
| 23 | Relative consumption rate | g g^-1^ h^-1^ |
| 24 | Fresh-weight consumption index | index |
| 25 | Relative assimilation rate | g g^-1^ h^-1^ |
| 26 | Approximate digestibility | g g^-1^ |
| 27 | Feeding rate | cm^3^ g^-1^ day^-1^ |
| 28 | Mean dry weight of grasshoppers | g degree-days^-1^ |
| 29 | Development | instar degree-days^-1^ |
| 30 | Mortality | % |
| 31 | Final dry weights of grasshoppers | mg |
| 32 | growth rate | mg day^-1^ |
| 33 | Consumption rate | mg day^-1^ |
| 34 | Efficiency of conversion of ingested matter | index |
| 35 | Efficiency of conversion of digested matter | index |
