# Supplementary material for: Should I Eat or Should I Go? Acridid Grasshoppers and Their Novel Host Plants: Potential for Biotic Resistance
Source: Plants (Basel). 2018 Oct 7;7(4):83. doi: 10.3390/plants7040083 (PMC6313845; doi:10.3390/plants7040083)
Supplement: Supplementary file 1 [file plants-07-00083-s001.zip › SM_revised2/TableS4.docx]

**Table S4.** Intermediate calculations of the effect size needed for the meta-analysis. Random-effects model.

| Record # | Outcome | SE | Var | W | W*es | W *(es^2^) | W^2^ | W_v_ | W_v_*es | W_v_*(es^2^) | W _v_ ^2^ |
| --- | --- | --- | --- | --- | --- | --- | --- | --- | --- | --- | --- |
| 1 | 0.333333 | 0.235702 | 0.055556 | 18 | 6 | 2 | 324 | 13.53427 | 4.511424 | 1.503808 | 183.1765 |
| 2 | 0.333333 | 0.235702 | 0.055556 | 18 | 6 | 2 | 324 | 13.53427 | 4.511424 | 1.503808 | 183.1765 |
| 3 | 0.333333 | 0.235702 | 0.055556 | 18 | 6 | 2 | 324 | 13.53427 | 4.511424 | 1.503808 | 183.1765 |
| 4 | 0.333333 | 0.235702 | 0.055556 | 18 | 6 | 2 | 324 | 13.53427 | 4.511424 | 1.503808 | 183.1765 |
| 5 | -0.09091 | 0.090909 | 0.008264 | 121 | -11 | 1 | 14641 | 37.60048 | -3.41823 | 0.310748 | 1413.796 |
| 6 | -0.2 | 0.2 | 0.04 | 25 | -5 | 1 | 625 | 17.14356 | -3.42871 | 0.685742 | 293.9016 |
| 7 | 0.5 | 0.353553 | 0.125 | 8 | 4 | 2 | 64 | 6.97686 | 3.48843 | 1.744215 | 48.67658 |
| 8 | 0.333333 | 0.235702 | 0.055556 | 18 | 6 | 2 | 324 | 13.53427 | 4.511424 | 1.503808 | 183.1765 |
| 9 | 0.333333 | 0.235702 | 0.055556 | 18 | 6 | 2 | 324 | 13.53427 | 4.511424 | 1.503808 | 183.1765 |
| 10 | 0.333333 | 0.235702 | 0.055556 | 18 | 6 | 2 | 324 | 13.53427 | 4.511424 | 1.503808 | 183.1765 |
| 11 | 0.333333 | 0.235702 | 0.055556 | 18 | 6 | 2 | 324 | 13.53427 | 4.511424 | 1.503808 | 183.1765 |
| 12 | 0.333333 | 0.235702 | 0.055556 | 18 | 6 | 2 | 324 | 13.53427 | 4.511424 | 1.503808 | 183.1765 |
| 13 | 0.333333 | 0.235702 | 0.055556 | 18 | 6 | 2 | 324 | 13.53427 | 4.511424 | 1.503808 | 183.1765 |
| 14 | 0.2 | 0.2 | 0.04 | 25 | 5 | 1 | 625 | 17.14356 | 3.428712 | 0.685742 | 293.9016 |
| 15 | 0.5 | 0.353553 | 0.125 | 8 | 4 | 2 | 64 | 6.97686 | 3.48843 | 1.744215 | 48.67658 |
| 16 | -0.5 | 0.5 | 0.25 | 4 | -2 | 1 | 16 | 3.726741 | -1.86337 | 0.931685 | 13.8886 |
| 17 | -0.11111 | 0.078567 | 0.006173 | 162 | -18 | 2 | 26244 | 40.81002 | -4.53445 | 0.503827 | 1665.458 |
| 18 | -0.22222 | 0.111111 | 0.012346 | 81 | -18 | 4 | 6561 | 32.59811 | -7.24402 | 1.609783 | 1062.637 |
| 19 | 0.25 | 0.25 | 0.0625 | 16 | 4 | 1 | 256 | 12.3715 | 3.092875 | 0.773219 | 153.054 |
| 20 | -0.16667 | 0.166667 | 0.027778 | 36 | -6 | 1 | 1296 | 21.68787 | -3.61465 | 0.602441 | 470.3637 |
| 21 | 0.272727 | 0.157459 | 0.024793 | 40.33333 | 11 | 3 | 1626.778 | 23.18876 | 6.324208 | 1.724784 | 537.7188 |
| 22 | 0.181818 | 0.128565 | 0.016529 | 60.5 | 11 | 2 | 3660.25 | 28.68628 | 5.215687 | 0.948307 | 822.9027 |
| 23 | 0.055556 | 0.055556 | 0.003086 | 324 | 18 | 1 | 104976 | 46.69109 | 2.593949 | 0.144108 | 2180.057 |
| 24 | 0.25 | 0.25 | 0.0625 | 16 | 4 | 1 | 256 | 12.3715 | 3.092875 | 0.773219 | 153.054 |
| 25 | 0.25 | 0.25 | 0.0625 | 16 | 4 | 1 | 256 | 12.3715 | 3.092875 | 0.773219 | 153.054 |
| 26 | 0.454545 | 0.203279 | 0.041322 | 24.2 | 11 | 5 | 585.64 | 16.76354 | 7.619792 | 3.463542 | 281.0164 |
| 27 | 0.181818 | 0.128565 | 0.016529 | 60.5 | 11 | 2 | 3660.25 | 28.68628 | 5.215687 | 0.948307 | 822.9027 |
| 28 | 0.125 | 0.125 | 0.015625 | 64 | 8 | 1 | 4096 | 29.44992 | 3.681241 | 0.460155 | 867.298 |
| 29 | 0.333333 | 0.149071 | 0.022222 | 45 | 15 | 5 | 2025 | 24.65899 | 8.219662 | 2.739887 | 608.0656 |
| 31 | 0.25 | 0.25 | 0.0625 | 16 | 4 | 1 | 256 | 12.3715 | 3.092875 | 0.773219 | 153.054 |
| 32 | 0.25 | 0.25 | 0.0625 | 16 | 4 | 1 | 256 | 12.3715 | 3.092875 | 0.773219 | 153.054 |
| 34 | 0.272727 | 0.157459 | 0.024793 | 40.33333 | 11 | 3 | 1626.778 | 23.18876 | 6.324208 | 1.724784 | 537.7188 |
| 35 | 0.181818 | 0.128565 | 0.016529 | 60.5 | 11 | 2 | 3660.25 | 28.68628 | 5.215687 | 0.948307 | 822.9027 |
| 37 | -0.5 | 0.5 | 0.25 | 4 | -2 | 1 | 16 | 3.726741 | -1.86337 | 0.931685 | 13.8886 |
| 38 | 0.5 | 0.5 | 0.25 | 4 | 2 | 1 | 16 | 3.726741 | 1.863371 | 0.931685 | 13.8886 |
| 39 | 0.5 | 0.5 | 0.25 | 4 | 2 | 1 | 16 | 3.726741 | 1.863371 | 0.931685 | 13.8886 |
| 40 | 0.090909 | 0.090909 | 0.008264 | 121 | 11 | 1 | 14641 | 37.60048 | 3.418225 | 0.310748 | 1413.796 |
| 41 | -0.16667 | 0.166667 | 0.027778 | 36 | -6 | 1 | 1296 | 21.68787 | -3.61465 | 0.602441 | 470.3637 |
| 43 | 0.5 | 0.5 | 0.25 | 4 | 2 | 1 | 16 | 3.726741 | 1.863371 | 0.931685 | 13.8886 |
| 44 | 0.333333 | 0.235702 | 0.055556 | 18 | 6 | 2 | 324 | 13.53427 | 4.511424 | 1.503808 | 183.1765 |
| 45 | 0.333333 | 0.235702 | 0.055556 | 18 | 6 | 2 | 324 | 13.53427 | 4.511424 | 1.503808 | 183.1765 |
| 46 | 0.454545 | 0.203279 | 0.041322 | 24.2 | 11 | 5 | 585.64 | 16.76354 | 7.619792 | 3.463542 | 281.0164 |
| 47 | 0.272727 | 0.157459 | 0.024793 | 40.33333 | 11 | 3 | 1626.778 | 23.18876 | 6.324208 | 1.724784 | 537.7188 |
| 48 | 0.333333 | 0.235702 | 0.055556 | 18 | 6 | 2 | 324 | 13.53427 | 4.511424 | 1.503808 | 183.1765 |
| 49 | 0.333333 | 0.235702 | 0.055556 | 18 | 6 | 2 | 324 | 13.53427 | 4.511424 | 1.503808 | 183.1765 |
| 50 | 0.333333 | 0.235702 | 0.055556 | 18 | 6 | 2 | 324 | 13.53427 | 4.511424 | 1.503808 | 183.1765 |
| 51 | 0.333333 | 0.235702 | 0.055556 | 18 | 6 | 2 | 324 | 13.53427 | 4.511424 | 1.503808 | 183.1765 |
| 52 | 0.181818 | 0.128565 | 0.016529 | 60.5 | 11 | 2 | 3660.25 | 28.68628 | 5.215687 | 0.948307 | 822.9027 |
| 54 | -0.16667 | 0.096225 | 0.009259 | 108 | -18 | 3 | 11664 | 36.24475 | -6.04079 | 1.006799 | 1313.682 |
| 55 | -0.2 | 0.2 | 0.04 | 25 | -5 | 1 | 625 | 17.14356 | -3.42871 | 0.685742 | 293.9016 |
| 58 | 0.181818 | 0.128565 | 0.016529 | 60.5 | 11 | 2 | 3660.25 | 28.68628 | 5.215687 | 0.948307 | 822.9027 |
| 59 | -0.16667 | 0.166667 | 0.027778 | 36 | -6 | 1 | 1296 | 21.68787 | -3.61465 | 0.602441 | 470.3637 |
| 60 | -0.22222 | 0.111111 | 0.012346 | 81 | -18 | 4 | 6561 | 32.59811 | -7.24402 | 1.609783 | 1062.637 |
| 62 | -0.5 | 0.5 | 0.25 | 4 | -2 | 1 | 16 | 3.726741 | -1.86337 | 0.931685 | 13.8886 |
| 63 | -0.11111 | 0.078567 | 0.006173 | 162 | -18 | 2 | 26244 | 40.81002 | -4.53445 | 0.503827 | 1665.458 |
|  |  |  |  |  |  |  |  |  |  |  |  |
| k | 55 |  | Sums: | 2331.9 | 162 | 106 | 254456.9 | 1036.801 | 125.5391 | 66.91275 | 25712.16 |
| df | 54 |  |  |  |  |  |  |  |  |  |  |
| Q | 94.74565805 |  |  |  |  |  |  |  |  |  |  |
| I^2^ | 43.00530376 |  |  |  |  |  | v | 0.018331 |  |  |  |
|  |  |  |  |  |  |  |  |  |  |  |  |
| Q_v_ | 51.71207618 |  | es(random) | 0.004075 |  |  |  |  |  |  |  |
| I_v_^2^ | -4.424351107 |  | Sees (random) | 0.001982 |  |  |  |  |  |  |  |
|  |  |  | CI (random) | 0.000189 | 0.00796 |  |  |  |  |  |  |
