# Supplementary material for: Should I Eat or Should I Go? Acridid Grasshoppers and Their Novel Host Plants: Potential for Biotic Resistance
Source: Plants (Basel). 2018 Oct 7;7(4):83. doi: 10.3390/plants7040083 (PMC6313845; doi:10.3390/plants7040083)
Supplement: Supplementary file 1 [file plants-07-00083-s001.zip › SM_revised2/TableS5.docx]

**Table S5**. Data for the forest plot

| Record # | Outcome | SE | CI lower | CI upper | Rate | CI100 lower | CI100 upper | Order in forest plot | Sample size, n |
| --- | --- | --- | --- | --- | --- | --- | --- | --- | --- |
| 1 | 0.333333 | 0.235702 | -0.12864 | 0.79531 | 33.33333 | 46.19764 | 112.8643 | 56 | 6 |
| 2 | 0.333333 | 0.235702 | -0.12864 | 0.79531 | 33.33333 | 46.19764 | 112.8643 | 55 | 6 |
| 3 | 0.333333 | 0.235702 | -0.12864 | 0.79531 | 33.33333 | 46.19764 | 112.8643 | 54 | 6 |
| 4 | 0.333333 | 0.235702 | -0.12864 | 0.79531 | 33.33333 | 46.19764 | 112.8643 | 53 | 6 |
| 5 | -0.09091 | 0.090909 | -0.26909 | 0.087273 | -9.09091 | 17.81818 | -0.36364 | 52 | 11 |
| 6 | -0.2 | 0.2 | -0.592 | 0.192 | -20 | 39.2 | -0.8 | 51 | 5 |
| 7 | 0.5 | 0.353553 | -0.19296 | 1.192965 | 50 | 69.29646 | 169.2965 | 50 | 4 |
| 8 | 0.333333 | 0.235702 | -0.12864 | 0.79531 | 33.33333 | 46.19764 | 112.8643 | 49 | 6 |
| 9 | 0.333333 | 0.235702 | -0.12864 | 0.79531 | 33.33333 | 46.19764 | 112.8643 | 48 | 6 |
| 10 | 0.333333 | 0.235702 | -0.12864 | 0.79531 | 33.33333 | 46.19764 | 112.8643 | 47 | 6 |
| 11 | 0.333333 | 0.235702 | -0.12864 | 0.79531 | 33.33333 | 46.19764 | 112.8643 | 46 | 6 |
| 12 | 0.333333 | 0.235702 | -0.12864 | 0.79531 | 33.33333 | 46.19764 | 112.8643 | 45 | 6 |
| 13 | 0.333333 | 0.235702 | -0.12864 | 0.79531 | 33.33333 | 46.19764 | 112.8643 | 44 | 6 |
| 14 | 0.2 | 0.2 | -0.192 | 0.592 | 20 | 39.2 | 79.2 | 43 | 5 |
| 15 | 0.5 | 0.353553 | -0.19296 | 1.192965 | 50 | 69.29646 | 169.2965 | 42 | 4 |
| 16 | -0.5 | 0.5 | -1.48 | 0.48 | -50 | 98 | -2 | 41 | 2 |
| 17 | -0.11111 | 0.078567 | -0.2651 | 0.042881 | -11.1111 | 15.39921 | -6.82301 | 40 | 18 |
| 18 | -0.22222 | 0.111111 | -0.44 | -0.00444 | -22.2222 | 21.77778 | -22.6667 | 39 | 18 |
| 19 | 0.25 | 0.25 | -0.24 | 0.74 | 25 | 49 | 99 | 38 | 4 |
| 20 | -0.16667 | 0.166667 | -0.49333 | 0.16 | -16.6667 | 32.66667 | -0.66667 | 37 | 6 |
| 21 | 0.272727 | 0.157459 | -0.03589 | 0.581347 | 27.27273 | 30.862 | 85.40745 | 36 | 11 |
| 22 | 0.181818 | 0.128565 | -0.07017 | 0.433805 | 18.18182 | 25.19871 | 61.56235 | 35 | 11 |
| 23 | 0.055556 | 0.055556 | -0.05333 | 0.164444 | 5.555556 | 10.88889 | 22 | 34 | 18 |
| 24 | 0.25 | 0.25 | -0.24 | 0.74 | 25 | 49 | 99 | 33 | 4 |
| 25 | 0.25 | 0.25 | -0.24 | 0.74 | 25 | 49 | 99 | 32 | 4 |
| 26 | 0.454545 | 0.203279 | 0.056119 | 0.852972 | 45.45455 | 39.84267 | 130.7518 | 31 | 11 |
| 27 | 0.181818 | 0.128565 | -0.07017 | 0.433805 | 18.18182 | 25.19871 | 61.56235 | 30 | 11 |
| 28 | 0.125 | 0.125 | -0.12 | 0.37 | 12.5 | 24.5 | 49.5 | 29 | 8 |
| 29 | 0.333333 | 0.149071 | 0.041154 | 0.625513 | 33.33333 | 29.21795 | 95.88462 | 28 | 15 |
| 31 | 0.25 | 0.25 | -0.24 | 0.74 | 25 | 49 | 99 | 27 | 4 |
| 32 | 0.25 | 0.25 | -0.24 | 0.74 | 25 | 49 | 99 | 26 | 4 |
| 34 | 0.272727 | 0.157459 | -0.03589 | 0.581347 | 27.27273 | 30.862 | 85.40745 | 25 | 11 |
| 35 | 0.181818 | 0.128565 | -0.07017 | 0.433805 | 18.18182 | 25.19871 | 61.56235 | 24 | 11 |
| 37 | -0.5 | 0.5 | -1.48 | 0.48 | -50 | 98 | -2 | 23 | 2 |
| 38 | 0.5 | 0.5 | -0.48 | 1.48 | 50 | 98 | 198 | 22 | 2 |
| 39 | 0.5 | 0.5 | -0.48 | 1.48 | 50 | 98 | 198 | 21 | 2 |
| 40 | 0.090909 | 0.090909 | -0.08727 | 0.269091 | 9.090909 | 17.81818 | 36 | 20 | 11 |
| 41 | -0.16667 | 0.166667 | -0.49333 | 0.16 | -16.6667 | 32.66667 | -0.66667 | 19 | 6 |
| 43 | 0.5 | 0.5 | -0.48 | 1.48 | 50 | 98 | 198 | 18 | 2 |
| 44 | 0.333333 | 0.235702 | -0.12864 | 0.79531 | 33.33333 | 46.19764 | 112.8643 | 17 | 6 |
| 45 | 0.333333 | 0.235702 | -0.12864 | 0.79531 | 33.33333 | 46.19764 | 112.8643 | 16 | 6 |
| 46 | 0.454545 | 0.203279 | 0.056119 | 0.852972 | 45.45455 | 39.84267 | 130.7518 | 15 | 11 |
| 47 | 0.272727 | 0.157459 | -0.03589 | 0.581347 | 27.27273 | 30.862 | 85.40745 | 14 | 11 |
| 48 | 0.333333 | 0.235702 | -0.12864 | 0.79531 | 33.33333 | 46.19764 | 112.8643 | 13 | 6 |
| 49 | 0.333333 | 0.235702 | -0.12864 | 0.79531 | 33.33333 | 46.19764 | 112.8643 | 12 | 6 |
| 50 | 0.333333 | 0.235702 | -0.12864 | 0.79531 | 33.33333 | 46.19764 | 112.8643 | 11 | 6 |
| 51 | 0.333333 | 0.235702 | -0.12864 | 0.79531 | 33.33333 | 46.19764 | 112.8643 | 10 | 6 |
| 52 | 0.181818 | 0.128565 | -0.07017 | 0.433805 | 18.18182 | 25.19871 | 61.56235 | 9 | 11 |
| 54 | -0.16667 | 0.096225 | -0.35527 | 0.021934 | -16.6667 | 18.86011 | -14.4732 | 8 | 18 |
| 55 | -0.2 | 0.2 | -0.592 | 0.192 | -20 | 39.2 | -0.8 | 7 | 5 |
| 58 | 0.181818 | 0.128565 | -0.07017 | 0.433805 | 18.18182 | 25.19871 | 61.56235 | 6 | 11 |
| 59 | -0.16667 | 0.166667 | -0.49333 | 0.16 | -16.6667 | 32.66667 | -0.66667 | 5 | 6 |
| 60 | -0.22222 | 0.111111 | -0.44 | -0.00444 | -22.2222 | 21.77778 | -22.6667 | 4 | 18 |
| 62 | -0.5 | 0.5 | -1.48 | 0.48 | -50 | 98 | -2 | 3 | 2 |
| 63 | -0.11111 | 0.078567 | -0.2651 | 0.042881 | -11.1111 | 15.39921 | -6.82301 | 2 | 18 |
| Effect summary | 0.004075 | 0.001982 | 0.000189 | 0.00796 | 0.407457 | 0.388552 | 1.203465 | 1 | 1 |
