# Supplementary figures and images for: Should I Eat or Should I Go? Acridid Grasshoppers and Their Novel Host Plants: Potential for Biotic Resistance
Source: Plants (Basel). 2018 Oct 7;7(4):83. doi: 10.3390/plants7040083 (PMC6313845; doi:10.3390/plants7040083)

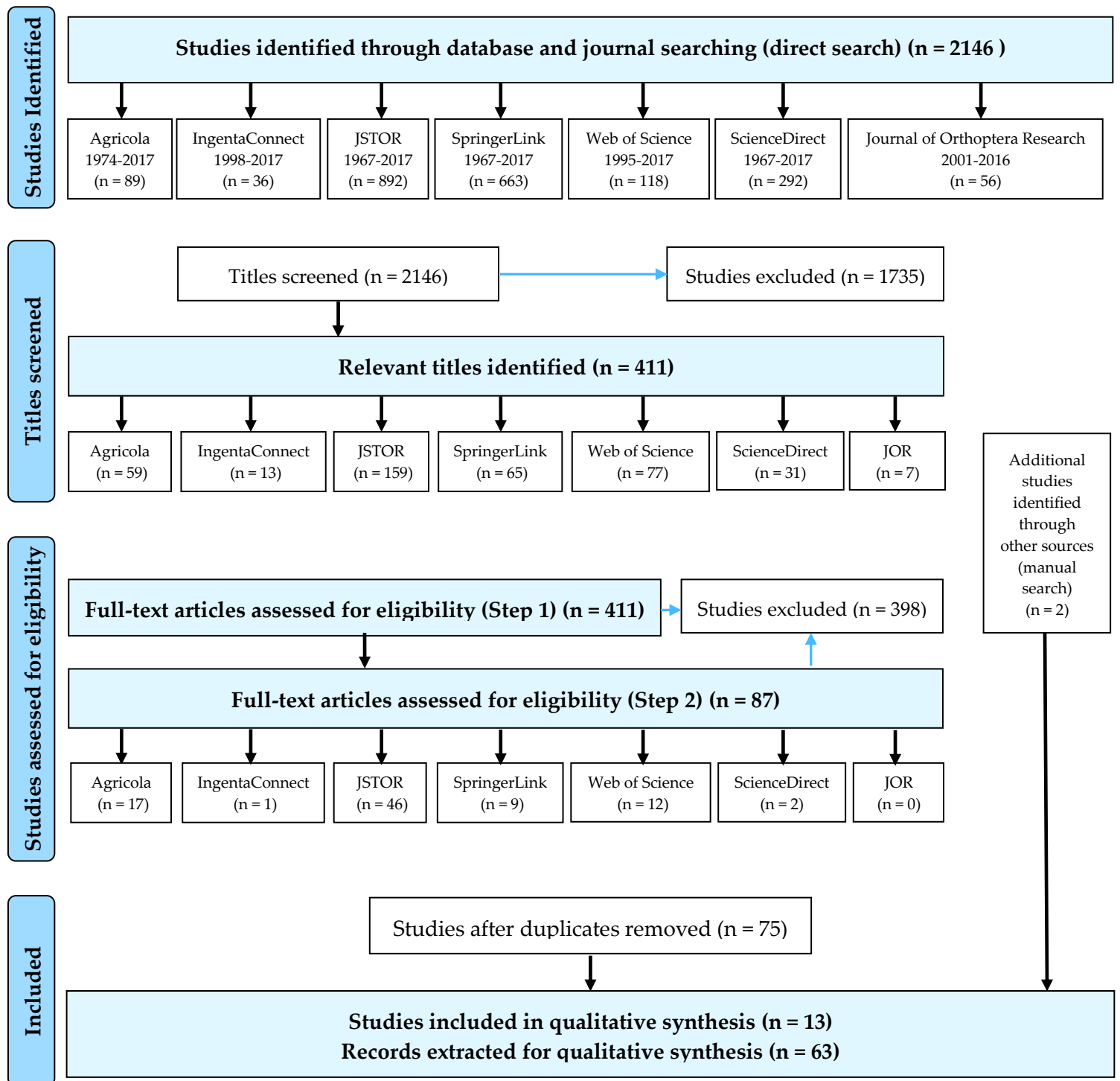

**Figure S1.** Literature search and data collection: PRISMA flowchart (*modified from Moher et al. [63]*).

Supplement: Supplementary file 1 [file plants-07-00083-s001.zip › SM_revised2/Fig S1.pdf]
